# Dynamic tracking of pathogenic receptor expression of live cells using pyrenyl glycoanthraquinone-decorated graphene electrodes†
†Electronic supplementary information (ESI) available: Experimental section, additional figures and original spectra copy of new compounds. See DOI: 10.1039/c4sc03614j



**DOI:** 10.1039/c4sc03614j

**Published:** 2015-01-13

**Authors:** Xiao-Peng He, Bi-Wen Zhu, Yi Zang, Jia Li, Guo-Rong Chen, He Tian, Yi-Tao Long

**Affiliations:** a Key Laboratory for Advanced Materials & Institute of Fine Chemicals , East China University of Science and Technology , 130 Meilong Rd. , Shanghai 200237 , PR China . Email: xphe@ecust.edu.cn ; Email: ytlong@ecust.edu.cn ; Fax: +86-21-64252758 ; Tel: +86-21-64253016; b National Center for Drug Screening , State Key Laboratory of Drug Research , Shanghai Institute of Materia Medica , Chinese Academy of Sciences , Shanghai 201203 , PR China . Email: jli@simm.ac.cn

## Abstract

Expression of specific transmembrane receptors by cells frequently represents an important signature of diseases, but this dynamic event can hardly be monitored directly with live cells due to the limitation of current biochemical techniques. Here we develop a pyrenyl glycoanthraquinone construct that can be firmly immobilized on a graphene-spotted screen printed electrode *via* strong π-interactions. The inherent current signal produced by the surface-confined glycoquinone can be used to detect selective sugar–protein recognitions with simple electrochemical techniques and portable facilities. Importantly, we demonstrate that the level of pathogenic receptors expressed by different types of live cells can be tracked with the electrode system in a label-free manner, providing a useful tool for the on-demand disease diagnosis as well as basic biochemical studies.

## Abstract 2

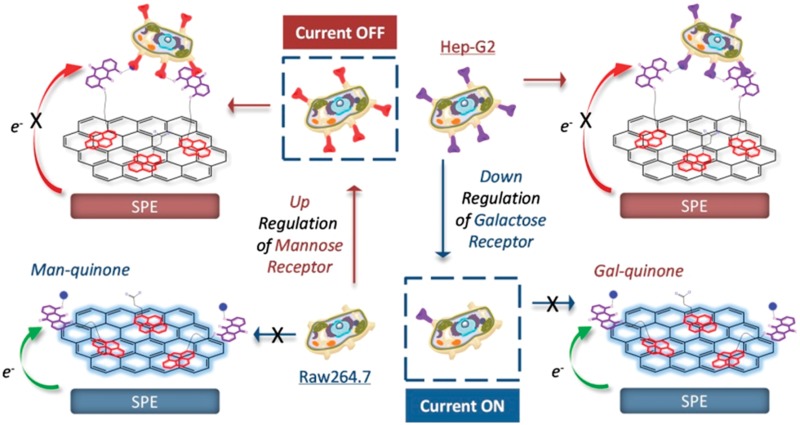
Dynamic tracking of pathogenic receptor expression with live cells is made possible by pyrenyl glycoanthraquinones decorated on graphene electrodes.

## Introduction

Cells express different levels and types of receptors on the cell membrane under distinct physio- and pathological conditions. The over-expression of certain transmembrane receptors is often a critical signature of diseases. For example, based on the microenvironment, macrophages may acquire diverse functional phenotypes. Two well-established polarized phenotypes are classically (M1) and alternatively activated (M2) macrophages. While M1 plays a positive role in destroying foreign organisms and tumor cells, M2 with over-expressed mannose receptors (MRs, CD206) may provoke pathological events such as chronic infections, tumorigenesis, and tumor metastasis.^1^ The asialoglycoprotein receptors (ASGPrs) are expressed predominantly on hepatocytes for clearance of galactose-terminated glycoproteins. Nevertheless, it is suggested that the ASGPrs can also serve as an entry site for hepatotropic viruses, and are over-expressed during liver inflammation.^2,3^ As a result, tracking of the pathogenic receptor expression of live cells may offer valuable insights into disease diagnosis as well as the advancement of cell biology.

Conventional techniques for detection of cell receptors require cell lysis, and the lysates are then subjected to sandwich-based immunoassays, which are intricate and time-consuming. While lysis of live cells might compromise the structure and, especially conformation of the receptor of interest, the cost of the immunoassay technique is high. These drawbacks could slow down or even obscure the profiling of a particular cell. Alternatively, a number of label-free (no need to label the analyte) techniques have been developed, which include surface plasmon resonance (SPR), quartz crystal microbalance (QCM), electric field effect (EFE)^4^ and the construction of fluorogenic composite materials (FCM).^5,6^ Despite the simplified detection procedures of these techniques, they possess several flaws in terms of the following factors: (1) bulky detection facilities are employed, limiting the potential application for on-demand diagnosis; (2) pre-derivatization of the working surface is required to covalently link a probe molecule, increasing the detection time and cost.

Electrochemistry is a solid-phase technique extensively employed in the field of bio-recognition. The essential merit of exploiting electrochemistry relies on the use of portable detection facilities (normally a diminutive workstation for recording the data connected to a personal laptop for reading the data), the ease in manipulation and its ultra-sensitivity against redox processes taking place on the electrode surface. These attributes are not only promising for on-demand diagnoses, but also for laboratory popularization. On the other hand, functionalization of a working electrode can be spontaneous (*e.g.* the gold electrode–alkenethiol self-assembly),^7^ largely diminishing the cost and time consumed for the sensor fabrication.

Recently, considering the high-cost related to the massive production of gold electrodes, alternative electrode materials have been explored. Graphene,^8^ owing to its good electric properties and cheapness, has evolved as a promising class of working electrode materials.^9–12^ Indeed, a number of graphene-based electrochemical and optical systems have been constructed for the detection of pathogenic receptors and biomarkers including thrombin, gp120, amyloid β, cyclin A2, caspase-3 and metalloprotease.^13,14^ Whereas the majority of previously developed graphene electrodes for biological detections depends on electrochemical impedance spectroscopy, which requires the presence of an additional solution-dispersed redox probe,^15–19^ development of more sophisticated sensor systems with an inherent signal output may further simplify the detection process. In particular, self-assembled graphene composite electrodes that can selectively probe dynamic cellular events directly with live cells have been elusive.

**Fig. 1:**
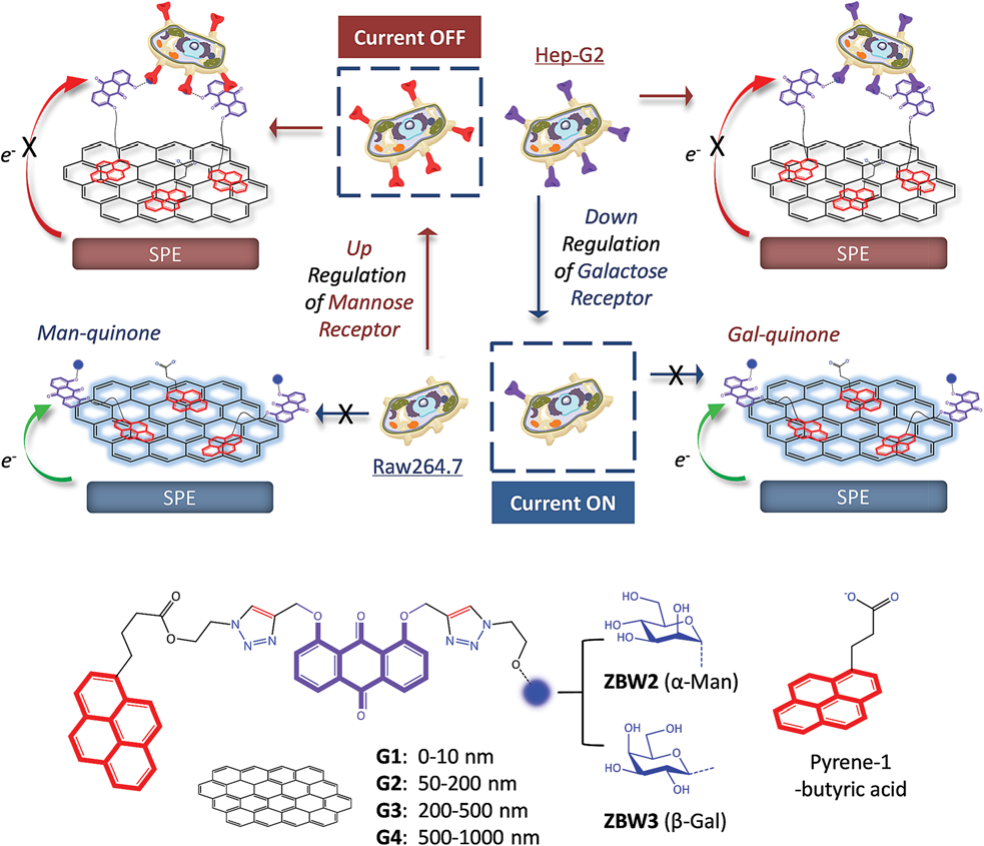
Cartoon depicting the dynamic tracking of up- or down-regulated pathogenic receptors expressed by different cells using pyrenyl mannosyl (Man) and galactosyl (Gal) anthraquinones decorated on graphene (**G1–G4** represent graphene oxides with different sizes)-spotted screen printed electrodes (SPEs). The electrodes are diluted with pyrene-1-butyric acid to facilitate multivalent sugar–receptor binding. The electron transfer (ET) of the electrode decorated with a glycoquinone is impeded (current OFF) once the specific receptor is over-expressed, and, reversely knockdown of the receptor leads to activated ET (current ON).

Here we have developed an integrated pyrenyl glycoquinone (GQ) construct that can be firmly immobilized onto graphene-spotted screen printed electrodes (SPEs) by strong π-interactions (Fig. 1). The resulting GQ-decorated graphene SPEs produce an intrinsic voltammetric signal of quinone, which makes possible the label-free detection of selective sugar–protein interactions. We demonstrate that the SPEs constructed have the ability to track the up- or down-regulated level of pathogenic receptors expressed by live cells using simple electrochemical techniques.

## Results and discussion

The target compounds were synthesized by coupling of a dipropargyl anthraquinone (AQ)^20^ with an azido glycoside, and then with an azido pyrene *via* a two-step copper(i)-catalyzed azide–alkyne cycloaddition (CuAAC) click chemistry. Azido β-galactoside and α-mannoside that can be recognized by the ASGPr expressed by hepatocytes^6,21–23^ and MR expressed by M2 macrophages^1^ were used to ‘click’ with AQ, respectively. Then, deacylation followed by the second ‘click’ with an azido pyrene gave the final products **ZBW2** and **ZBW3** (Fig. 1, Scheme S1 and S2†). To test the impact of chain length on the resulting sensing performance, **ZBW2′** (Scheme S3†) with a longer triethylene glycol linkage that connects between the pyrene and anthraquinone moieties was prepared by the same synthetic protocol.

With the compounds in hand, the graphene-spotted SPEs were fabricated. In former investigations as regards graphene-based biosensors, the graphene *size* effect has been barely probed. However, a recent study suggested that graphenes with different size might have distinct sensing properties.^24^ As a consequence, we used four commercially available graphene oxides (GOs) of different sizes (**G1**: 0–10 nm, **G2**: 50–200 nm, **G3**: 200–500 nm and **G4**: 500–1000 nm, Fig. 1) to optimize the sensor formation with SPEs made according to a previous study.^25^ The electrochemical processes taking place on the working electrode of SPE were interpreted with a portable workstation linked to a personal laptop.

Since the graphene SPEs were made succinctly by spotting a drop of (4 μL) graphene aqueous solution to the graphite working electrode area of SPE, GO that can be much more easily dispersed in water than the pristine graphene was used. The conventional electrochemical reduction of the surface-attached GOs to reduced-GOs was not carried out due to the following reasons: (1), the reduction will cause a substantial range of defect areas, decreasing the adsorbability for pyrene molecules, and (2) to block this defect, repeated spotting/reduction processes must be performed, which increases considerably the workload and cost of the sensor fabrication.^9–11^

Primarily, by spotting an identical amount of **ZBW2** (as a model to optimize the detection conditions, Fig. S1 and S2†) to the electrodes with GOs of different sizes, we observed that **G3** (200–500 nm) produced the largest current intensity (Fig. S1a†). This suggests that the graphene size effect might impact the adsorption of aromatic compounds, which is in agreement with a previous study.^24^ By using the **G3**-spotted electrodes, we further determined that the optimal GO and compound concentrations used were 0.5 mg mL^–1^ (Fig. S1b†) and 1 mM (Fig. S1c†), respectively.

Next, the bio-sensitivity of **ZBW2@G3** (mannosyl **ZBW2** on **G3**)-decorated electrodes was tested using a mannose/glucose-selective plant lectin, concanavalin A (Con A), by differential pulse voltammetry (DPV). It is envisioned that the specific sugar–lectin interactions may quench the current signal of AQ because of the encapsulation of the electroactive glycoquinone by the protein, blocking the electron transfer.^7,21^ We observed that the **G3**-spotted SPE similarly gave the best quenching effect ((*I*_0_ – *I*)/*I*_0_, where *I* and *I*_0_ are the current intensity in the presence and absence of 10 μM Con A, respectively) (Fig. S1d†). It has been reported that dense display of glycosides at a biomimetic interface may weaken its interaction with a specific lectin due to the intermolecular clustering effect.^26^ In contrast, flexible distribution of the glycosides may facilitate multivalent, high-avidity contacts with lectin matrices.^27^ As a result, pyrene-1-butyric acid was used to dilute the **ZBW2@G3** surface, and the optimal dilution ratio was determined to be 3/7 (**ZBW2**/pyrene-1-butyric acid) for Con A detection (Fig. S1e†). Eventually, **ZBW2′** with a longer PEG linkage between pyrene and AQ was used to test whether the chain elongation would impact the detection. The result shown in Fig. S1f† proved that **ZBW2@G3** with the shorter linkage had better sensitivity than **ZBW2′** in the presence of increasing Con A.

**Fig. 2:**
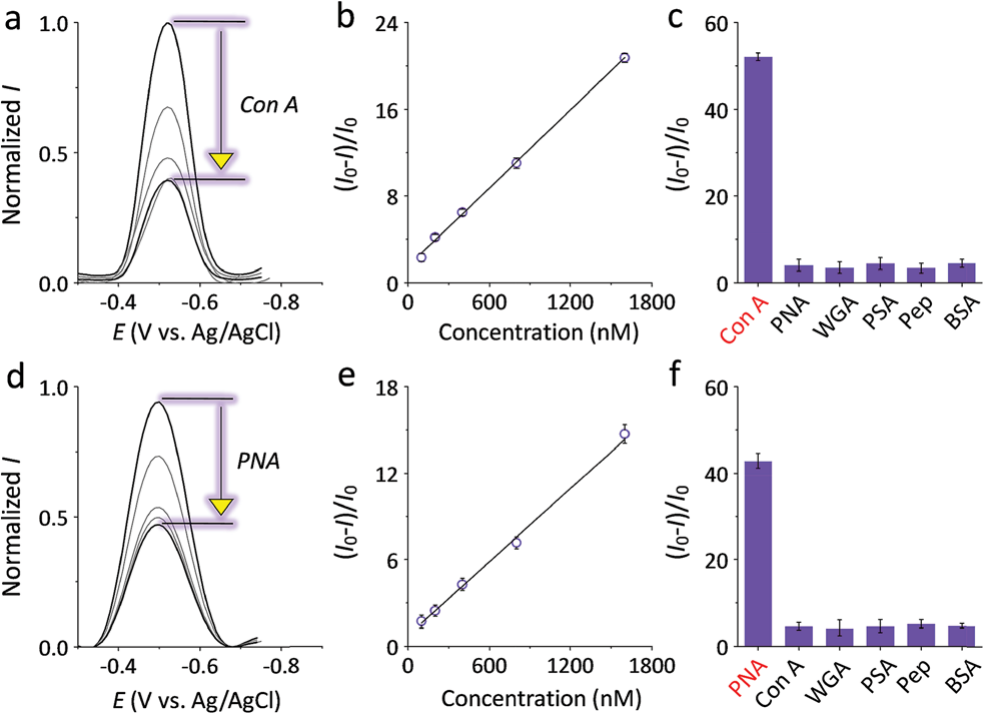
DPVs of SPEs decorated with (a) **ZBW2@G3** and (d) **ZBW3@G3** in the absence (the top curves) and presence of increasing specific lectin (concentration from the second top to bottom curve: 5, 10, 15 and 20 μM). Plotting of lectin sensitivity ((*I* – *I*_0_)/*I*_0_, where *I* and *I*_0_ are the current intensity in the presence and absence of a specific lectin, respectively) of (b) **ZBW2@G3** and (e) **ZBW3@G3** as a function of lectin concentration. Current quenching rate of (c) **ZBW2@G3** and (f) **ZBW3@G3** in the presence of 10 μM of different proteins. All DPVs were measured in Tris–HCl buffer (pH 7.3). Original DPVs of (c) and (f) are shown in Fig. S3a and S3b,† respectively.

With the optimized condition, the sensitivity and selectivity of the **G3** SPEs decorated with mannosyl **ZBW2** and galactosyl **ZBW3** was probed by a range of proteins. The presence of increasing Con A and peanut agglutinin (PNA, a galactose-selective lectin) led to gradual current quenching of **ZBW2@G3** (Fig. 2a) and **ZBW3@G3** (Fig. 2d), respectively. Good linearity for the plant lectin detection was produced over the nanomolar to sub-micromolar range (Fig. 2b for **ZBW2@G3** and Fig. 2e for **ZBW3@G3**), and the limit of detection (LOD) of **ZBW2@G3** (for Con A) and **ZBW3@G3** (for PNA) was determined to be 49 and 69 nM, respectively (3*σ*_b_/*k*, where *σ*_b_ is the current intensity in the absence of a lectin). In contrast, the presence of unselective lectins and proteins (Fig. 2c and f) including wheat germ agglutinin (WGA), *Pisum sativum* agglutinin (PSA), pepsin (Pep) and bovine serum albumin (BSA), and a range of physiological ions (Fig. S3d and S3e†) did not cause substantial variation of the current signal, suggesting the good selectivity of the SPEs constructed. We also synthesized a glucosyl pyrenyl anthraquinone **ZBW4** to test the biospecificity of the electrode system (Scheme S1 and S2†). We determined that **ZBW4@G3** showed sharp current quenching in the presence of Con A that is selective to both mannose and glucose^7^ with insignificant response to other lectins and proteins tested (Fig. S3c†).

To investigate whether the signal variation was a result of lectin adhesion to the pyrene-supported construct on graphene, composition between the compounds and **G3** was characterized. In the Raman spectra (Fig. S4a†), we observed that **ZBW2@G3** (0.90) and **ZBW3@G3** (0.89) showed increased *I*_D_/*I*_G_ (the intensity of D band (1355 cm^–1^)/G band (1600 cm^–1^)) ratio comparing to that of bare **G3** (0.85), suggesting the increase in carbon sp^2^-hybridization of the composite systems due to stacking of the compounds to graphene.^6,21,28,29^ The material composites also showed apparent red shifts in the UV-vis spectra (Fig. S4b† for **ZBW2@G3** (from 395 of the blue curve to 410 nm of the red curve) and Fig. S4c† for **ZBW3@G3** (from 410 nm of the blue curve to 420 nm of the red curve)) with respect to the compounds alone, which are indicative of π-stacking. In the meanwhile, peaks (*ν̃* = 2180 cm^–1^) characteristic of π-stacking were shown in the Fourier transform infrared spectra of the composites (Fig. S5†).^6,21,28,29^

**Fig. 3:**
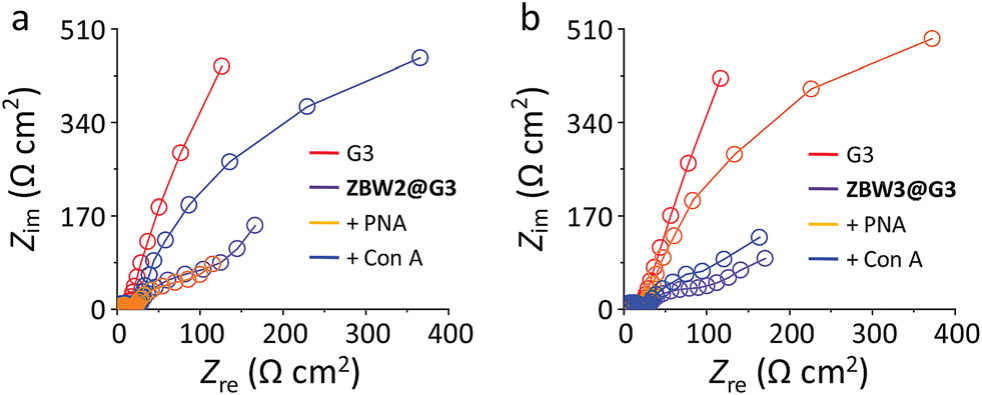
Nyquist plots of SPEs decorated with (a) **ZBW2@G3** and (b) **ZBW3@G3** in the absence and presence of selective or unselective lectin. Circuit models used to fit these Nyquist plots are shown in Fig. S6.†

**Fig. 4:**
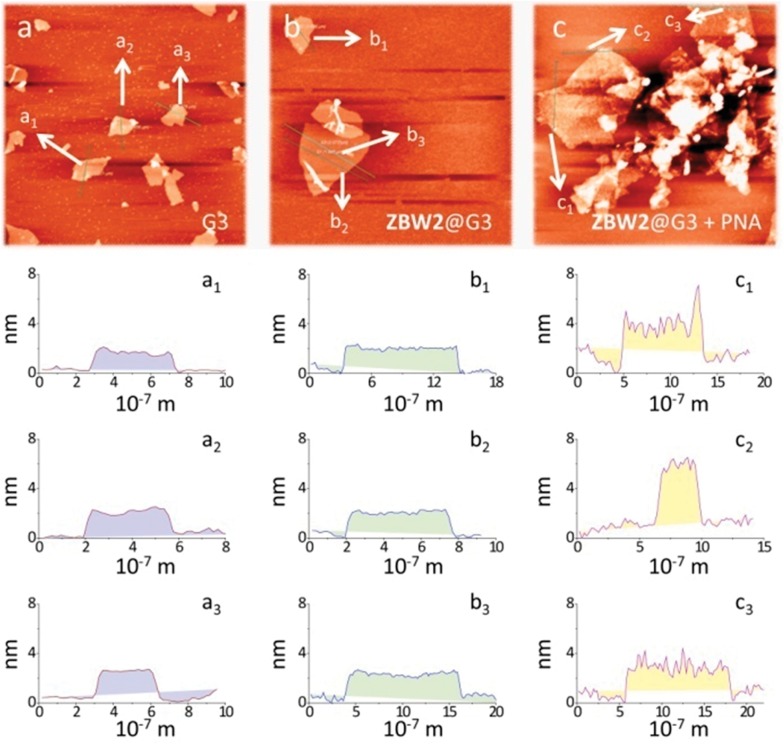
Atomic force microscope images of (a) **G3**, (b) **ZBW3@G3** and (c) **ZBW3@G3** in the presence of PNA.

Electrochemical impedance spectroscopy (EIS) was then employed to investigate the lectin adhesion on the electrodes using [Fe(CN)_6_]^3–/4–^ as the redox probe. We observed that, whereas addition of unselective lectin hardly caused any signal variation, the presence of a selective lectin led to sharp increment in the capacitive loop (increased charge resistibility) of the SPEs (Fig. 3a for **ZBW2@G3** and Fig. 3b for **ZBW3@G3**). This probably suggests the adhesion of the lectin to the glycoside-decorated electrode by forming sugar–lectin complexes, which is in good agreement with previous observations.^17–19^ To further demonstrate the complexation, we employed an atomic force microscope (AFM) to visualize the height increment of the composites. As shown in Fig. 4, while bare **G3** had an average height of *ca.* 1.80 nm (Fig. 4a), stacking of **ZBW3** increased the height to *ca.* 2.18 nm (Fig. 4b). Interestingly, addition of PNA to **ZBW3@G3** increased the height sharply to 4.0–7.0 nm (Fig. 4c), and this height increment accords well with the typical length of the lectin (about 3.2 nm)^30^ and with previous observations on sugar–lectin complexation at a carbon material interface.^31,32^ These data together suggest that the current quenching was probably a result of lectin adhesion onto the surface, blocking the electron transfer.

**Fig. 5:**
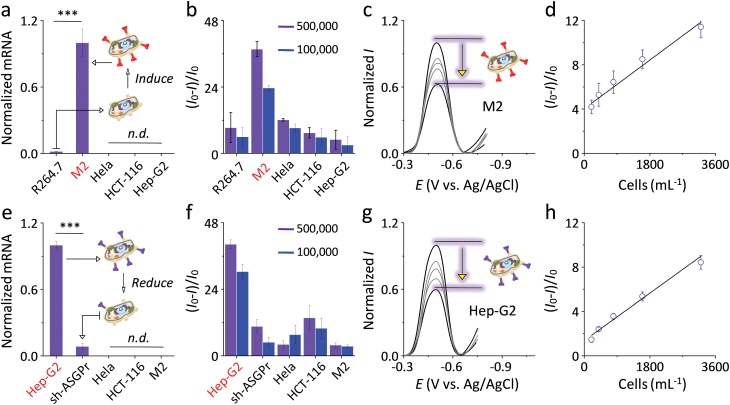
Normalized mRNA level of (a) macrophage mannose receptor (MR) and (e) asialoglycoprotein receptor (ASGPr) of various kinds of cells (n.d. means not detectable; ***P < 0.001). Plotting of current intensity change ((*I* – *I*_0_)/*I*_0_, where *I* and *I*_0_ are the current intensity in the presence and absence of cells, respectively) of (b) **ZBW2@G3** and (f) **ZBW3@G3** as a function of different types of cells. Normalized current intensity *I* in the presence of increasing (c) M2 cells with induced MR and (g) Hep-G2 cells with over-expressed ASGPr (from top to bottom: 0, 5 000, 10 000, 100 000 and 500 000 cells per mL). Plotting the current intensity change as a function of the concentration of (d) M2 cells and (h) Hep-G2 cells. Original DPVs of (b) and (f) are shown in Fig. S7a and b, and S7d and e,† respectively.

Next, the ability of the **G3** SPEs decorated with the pyrenyl glycoquinones to track pathogenic receptor expression of live cells was interrogated. Human Hep-G2 cells that over-express the galactose-selective ASGPr (which facilitates viral invasion and inflammation)^2,3^ and mouse RAW264.7 (R264.7) cells that can be induced to M2 macrophages with highly expressed MR (which promotes tumorigenesis and tumor metastasis)^1^ were employed. To realize the tracking of the dynamic receptor expression, knockdown of the ASGPr-1 gene of Hep-G2 (down-regulation of the galactose-receptor)^6,22^ and induction of the M2 macrophages by treatment of R264.7 with interleukin-4 (IL-4) (up-regulation of the mannose-receptor)^33^ were carried out. HeLa (human cervix cancer) and HCT-116 (human colon cancer) derived from different human tissues were used as negative controls. Quantification of the MR mRNA (Fig. 5a) by real-time quantitative polymerase chain reaction (PCR) indicated that the IL-4-induced M2 macrophages possess much higher MR expression level than raw R264.7 cells, and that Hep-G2, HeLa and HCT-116 cells do not express the receptor. PCT results of ASGPr1 mRNA (Fig. 5e) showed that silencing (sh) of ASGPr mRNA led to largely reduced receptor expression of sh-ASGPr cells, and that ASGPr is not expressed by HeLa, HCT-116 and M2 cells.

Subsequently, these live cells with or without expression of pathogenic receptors were incubated with the SPEs decorated with **ZBW2@G3** and **ZBW3@G3** with two cellular concentrations (100 000 and 500 000 cells per mL). On the one hand, while R264.7 macrophages without MR expression did not cause substantial current variation of the mannosyl **ZBW2@G3** (Fig. 5b), M2 macrophages with highly expressed MR caused evident, concentration-dependent (Fig. 5c) current quenching of the electrode with an LOD of 431 cells per mL (Fig. 5d). On the other hand, incubation of Hep-G2 with the galactosyl **ZBW3@G3** produced sharp (Fig. 5f) and gradual (Fig. 5g) current quenching of the electrode with an LOD of 248 cells per mL (Fig. 5h), whereas the presence of sh-ASGPr cells with largely reduced galactose receptors caused trivial current decrease.

Then, a competition assay was conducted by pre-incubation of M2 and Hep-G2 with increasing free d-mannose and d-galactose, respectively. We observed that the presence of the monosaccharides inhibited the current quenching of the two SPEs in a concentration-dependent manner (Fig. S7c for **ZBW2@G3** and Fig. S7f† for **ZBW3@G3**), suggesting that the detection was based on sugar–receptor interactions. More interestingly, both SPEs showed minute current change upon incubation with the cells that do not express the specific pathogenic receptors (Fig. 5b: mannosyl **ZBW2@G3** with HeLa, HCT-116 and Hep-G2; Fig. 5f: galactosyl **ZBW3@G3** with HeLa, HCT-116 and M2). These data suggest that the variation of pathogenic receptor expression of live cells of different kinds can be tracked by the glycoquinone-decorated graphene SPEs. The biocompatibility of the compounds and their GO composites was tested by the CCK-8 cytotoxicity assay.^34^ The results showed that **ZBW2**, **ZBW3** and their **G3** composites have little toxicity for Hep-G2 (Fig. S8†), which accords with previous observations that graphene-based electrodes are biocompatible materials for protein and live cell detection.^35–37^

## Conclusions

To summarize, we have developed a unique graphene electrode material spotted with a pyrenyl glycoquinone construct. The intrinsic voltammetric signal produced by the glycoquinones decorated on graphene could be exploited to detect selective sugar–lectin interactions in a label-free manner. Importantly, we showed that the electrodes had the ability to track the level of pathogenic receptor expression by different types of live cells by simple electrochemical techniques and portable facilities. This study may provide insight into the development of portable and low-cost devices suitable for the tracking of dynamic cellular events with live cells, facilitating the study of cell biology and early-stage clinical diagnoses.
