# Supplementary material for: Dynamic tracking of pathogenic receptor expression of live cells using pyrenyl glycoanthraquinone-decorated graphene electrodes
Source: Chem Sci. 2015 Jan 13;6(3):1996–2001. doi: 10.1039/c4sc03614j (PMC5496384; doi:10.1039/c4sc03614j)

Electronic Supplementary Material (ESI) for Chemical Science.
This journal is © The Royal Society of Chemistry 2015

Supporting Information

Dynamic tracking of pathogenic receptor expression of live cells using pyrenyl glycoanthraquinone-decorated graphene electrodes

Xiao-Peng He,[1]* Bi-Wen Zhu,[1] Yi Zang,[2] Jia Li,[2]* Guo-Rong Chen,[1] He Tian[1] and Yi-Tao Long[1]*

[1]*Key Laboratory for Advanced Materials & Institute of Fine Chemicals, East China University of Science and Technology, 130 Meilong Rd., Shanghai 200237, PR China*

[2]*National Center for Drug Screening, State Key Laboratory of Drug Research, Shanghai Institute of Materia Medica, Chinese Academy of Sciences, 189 Guo Shoujing Rd., Shanghai 201203, P. R. China*

*Corresponding authors' email:

xphe@ecust.edu.cn (X.-P. He)

jli@simm.ac.cn (J. Li)

ytlong@ecust.edu.cn (Y.-T. Long)

Contents list:

S1. Additional figures

S2. Experimental section (including Scheme S1-S3)

S3. NMR copy of new compounds

S1. Additional figures

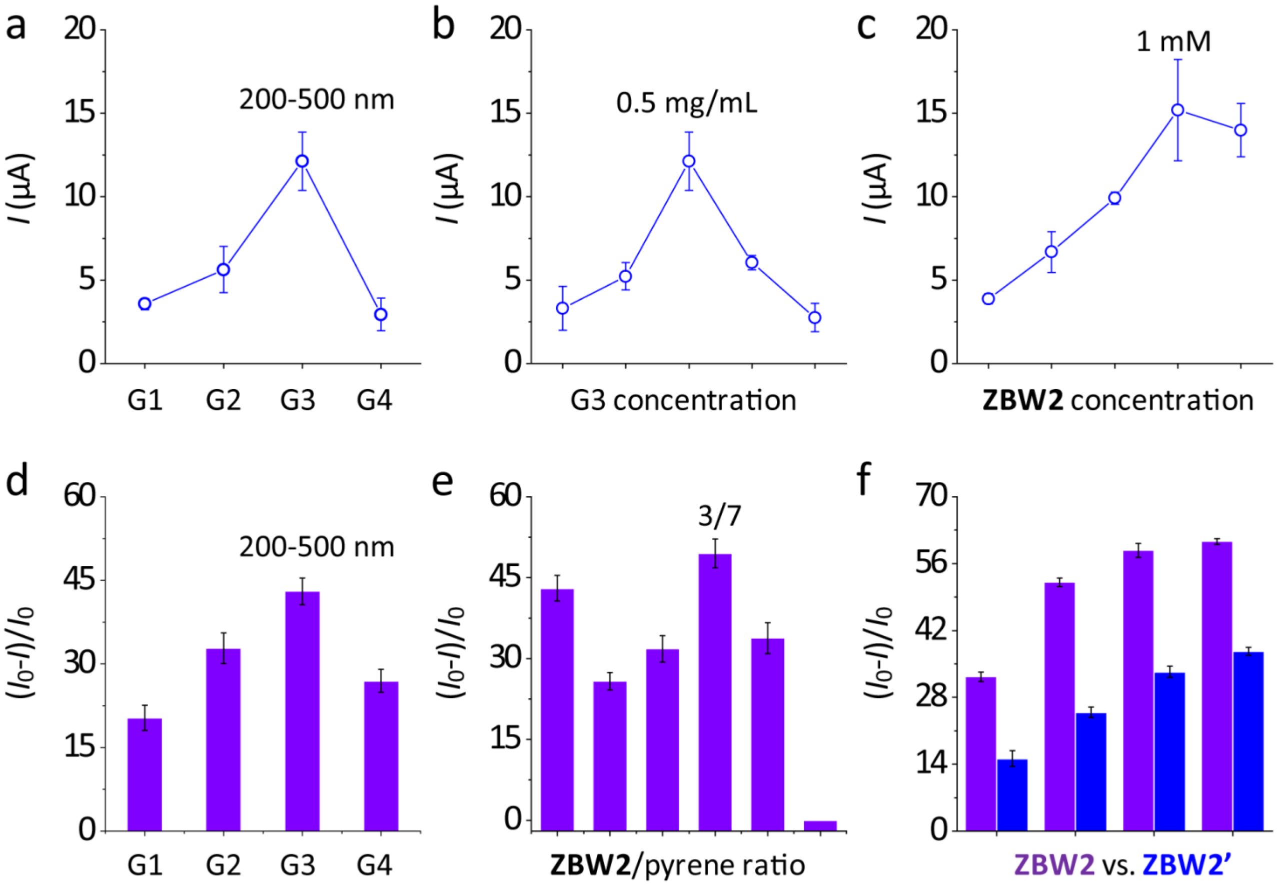

Figure S1. Plotting of current intensity (I) of **ZBW2** as a function of (a) graphene oxide (GO) of different sizes: **G1** (0-10 nm), **G2** (50-200 nm), **G3** (200-500 nm) and **G4** (500-1000 nm), (b) different **G3** concentrations (from left to right: 0.1, 0.3, 0.5, 0.8 and 1.0 mg/mL), and (c) different **ZBW2** concentrations (from left to right: 0.3, 0.5, 0.8, 1.0 and 2.0 mM). Plotting of lectin sensitivity ((I-I_0)/I_0, where I and I_0 are the current intensity in the presence and absence of 10 μM Con A, respectively) as a function of (d) **G1**-**G4**, (e) molar ratio of **ZBW2**/pyrene-1-butyric acid (from left to right: 10/0, 8/2, 5/5, 2/8 and 0/10), and (f) **ZBW2** (violet) and **ZBW2'** (blue) in the presence of increasing Con A (from left to right: 5, 10, 15, 20 μM). All DPVs were measured in Tris-HCl buffer (pH 7.3), and for all the original DPV plots, see Fig. S2 below.

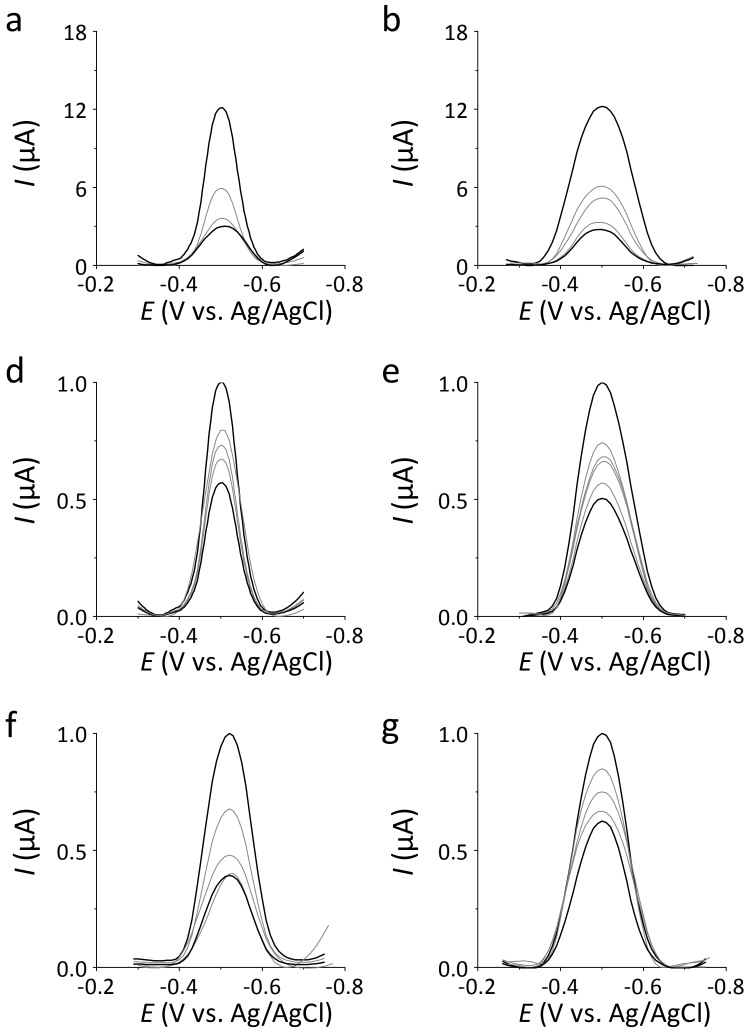

Figure S2. DPVs of SPE spotted with (a) **ZBW2** with graphene oxide (GO) of different sizes: **G1** (0-10 nm), **G2** (50-200 nm), **G3** (200-500 nm) and **G4** (500-1000 nm), (b) **ZBW2** with **G3** of different concentrations (0.1, 0.3, 0.5, 0.8 and 1.0 mg/mL), and (c) **ZBW2** with concentrations (0.3, 0.5, 0.8, 1.0 and 2.0 mM) on **G3**. The normalized DPVs of SPEs confined with (d) **ZBW2** (1 mM) on GO (0.5 mg/mL) of different sizes (from second top to bottom curve: **G1**, **G4**, **G2**, **G3**) in the absence (top curve) or presence of 10 μM Con A, (e) different **ZBW2**/pyrene-1-butyric acid ratios (from second top to bottom curve: 7/3, 5/5, 2/8, 10/0, 3/7) on **G3** in the absence (top curve) or presence of 10 μM Con A, and (f) **ZBW2@G3** and (g) **ZBW2'@G3** in the absence (top curve) and presence of increasing Con A (from second top to bottom: 5, 10, 15 and 20 μM).

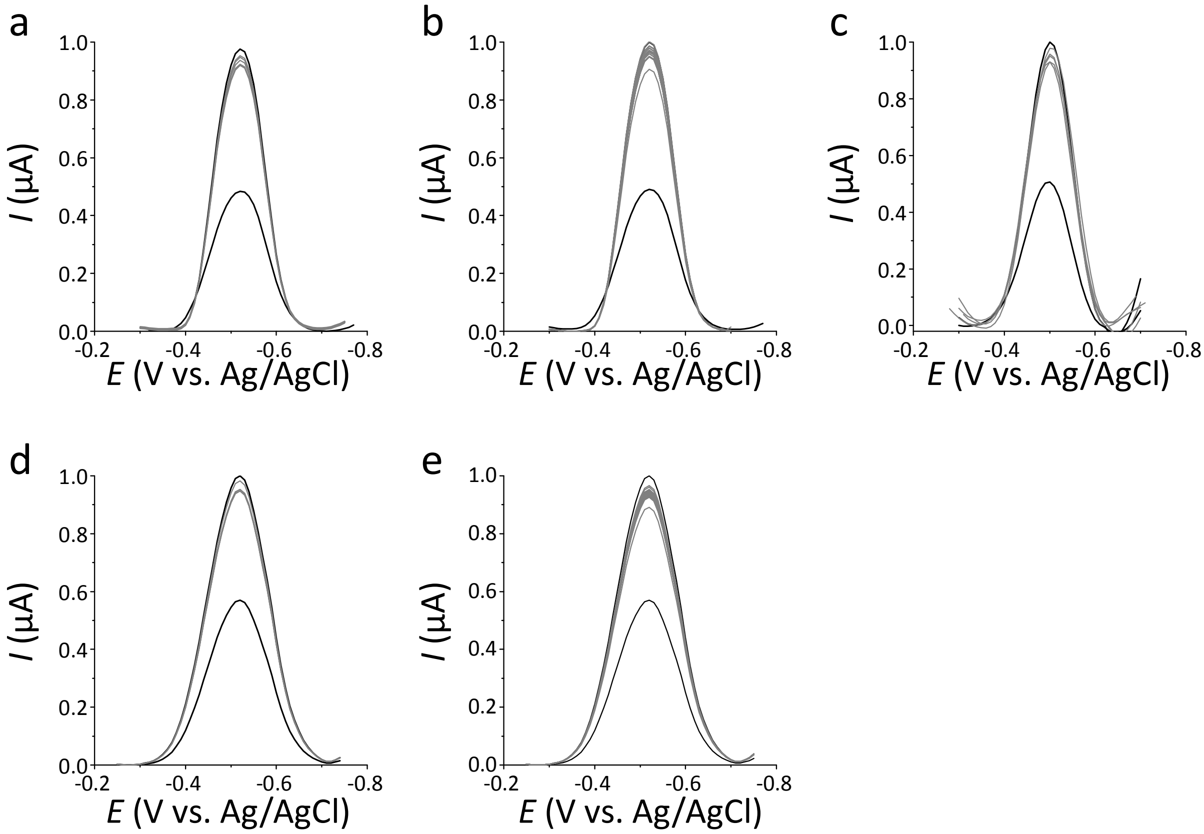

Figure S3. DPVs of (a) **ZBW2@G3** in the absence (top curve) and presence of 10 μM of various proteins (from top to bottom, BSA, PSA, PNA, WGA, Pep and Con A), (b) **ZBW3@G3** in the absence (top curve) and presence of 10 μM of various proteins (from top to bottom, WGA, Con A, BSA, PSA, Pep and PNA), (c) **ZBW4@G3** in the absence (top curve) and presence of 10 μM of various proteins (from top to bottom, BSA, PSA, PNA, WGA, Pep and Con A), (d) **ZBW2@G3** in absence (top curve) and presence of 10 mM of various ions (Ni^{+}, Co^{3+}, Na^{+}, Cr^{3+}, Fe^{2+}, K^{+}, Mg^{2+}, Cu^{2+}, Ca^{2+}, Mn^{2+}, Cl^{-}, Br^{-}, F^{-}, HCO_3^{-}, CO_3^{2-}, SO_4^{2-}, HSO_4^{-}, PO_4^{3-}, $HPO4^{2-}$, $H_2PO_2^{-}$, OH^{-}, NO_2^{-}) and 10 μM Con A (bottom curve), and (e) **ZBW3@G3** in absence (top curve) and presence of 10 mM of the various ions and 10 μM PNA (bottom curve). The current intensities (*I*) are normalized.

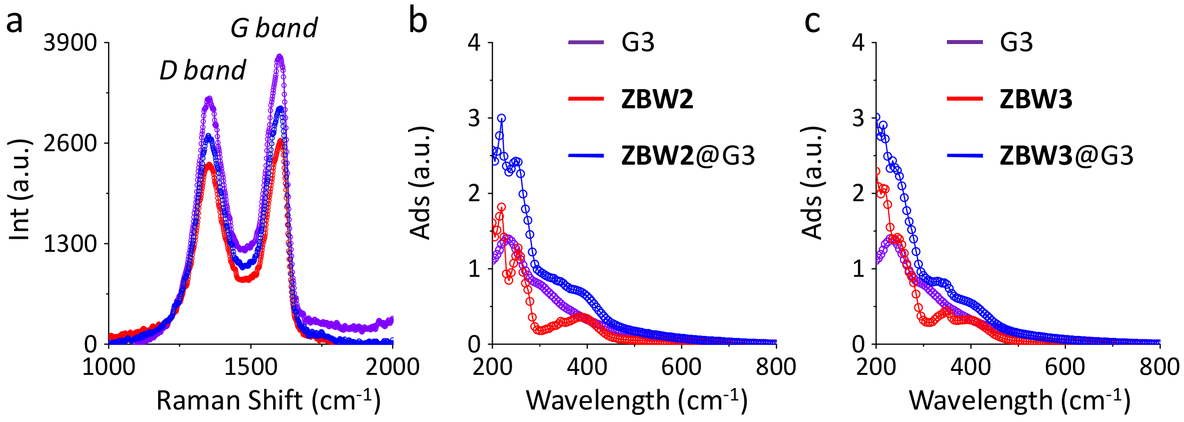

Figure S4. (a) Stacked Raman spectra of **G3** (violet), **ZBW2@G3** (red), and **ZBW3@G3** (blue) composite. UV spectra of (b) **G3**, **ZBW2**, and **ZBW2@G3**, and (c) **G3**, **ZBW3**, and **ZBW3@G3**.

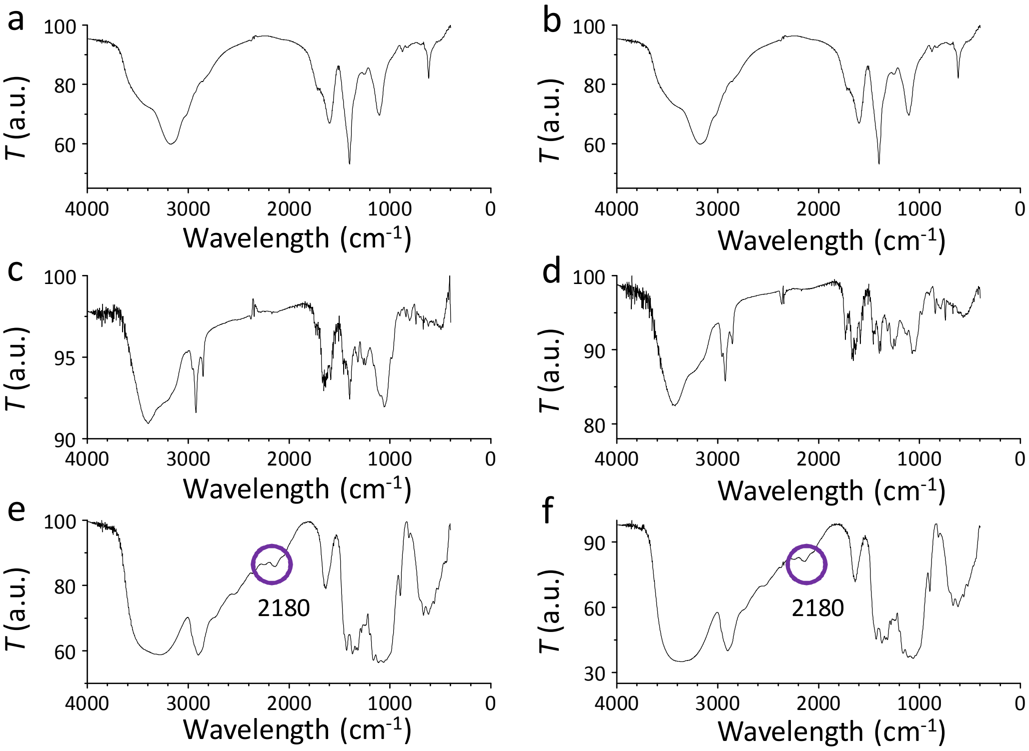

Figure S5. FTIR spectra of (a,b) **G3**, (c) **ZBW2**, (d) **ZBW3**, (e), **ZBW2@G3** and (f) **ZBW3@G3**. The broad band around 3200 cm^{-1} in the spectra can be mainly ascribed to the combination of the polar groups in GO with water molecules. The new band at 2840 cm^{-1} (C-H stretch of saturation chains) and 1350-1250 cm^{-1} (C-N stretch of aromatic nucleus) in (e,f) indicate the composition of compound onto the GO surface. The intensified peak at 2180 cm^{-1} are probably attributed to the π-π interaction between pyrene or anthraquinone with GO.

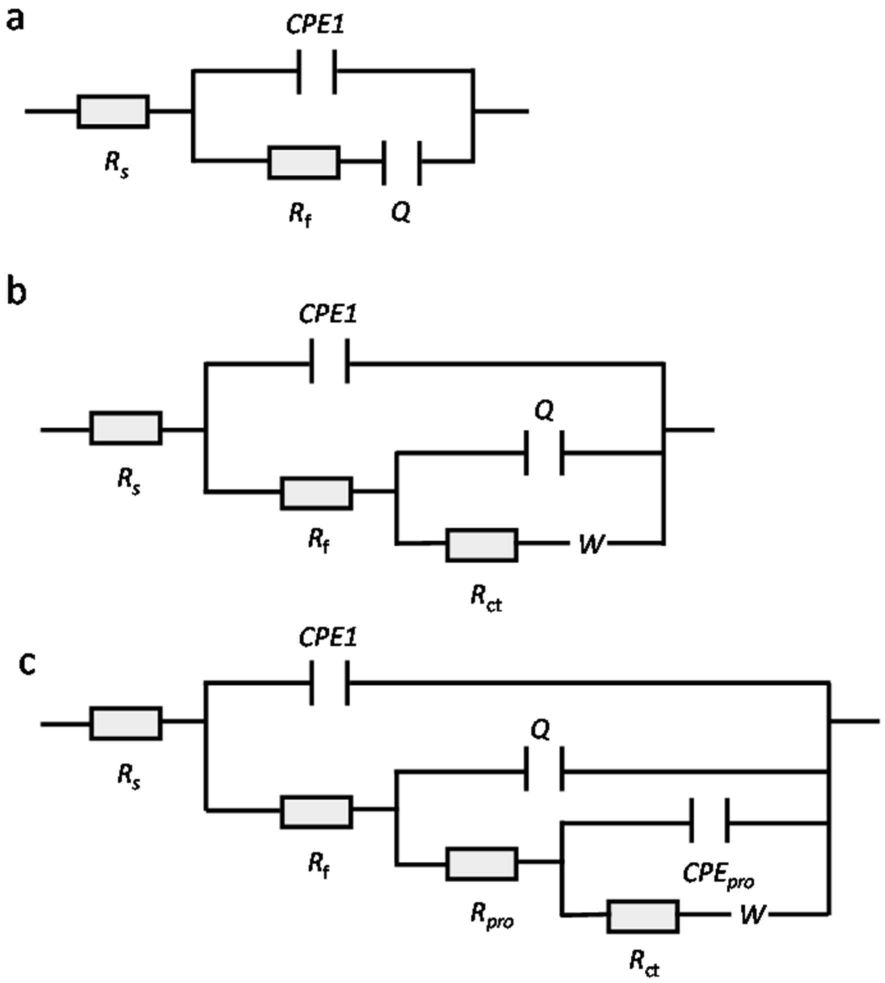

Figure S6. The electric circuit models used to fit the Nyquist plots of (a) SPEs doped with **G3**, (b) SPEs doped with **ZBW2@G3** and **ZBW3@G3**, and (c) SPEs doped with **ZBW2@G3** + Con A and **ZBW3@G3** + PNA, where R_s is the solvent resistance, R_f the film resistance, R_{ct} the charge transfer resistance, R_{pro} the protein resistance, *CPE* the constant phase element, *CPE1* the film capacitance, *Q* the double layer capacitance, CPE_{pro} the protein capacitance, and *W* the Warburg diffusion impedance. The experimental and simulated data are in good agreement.

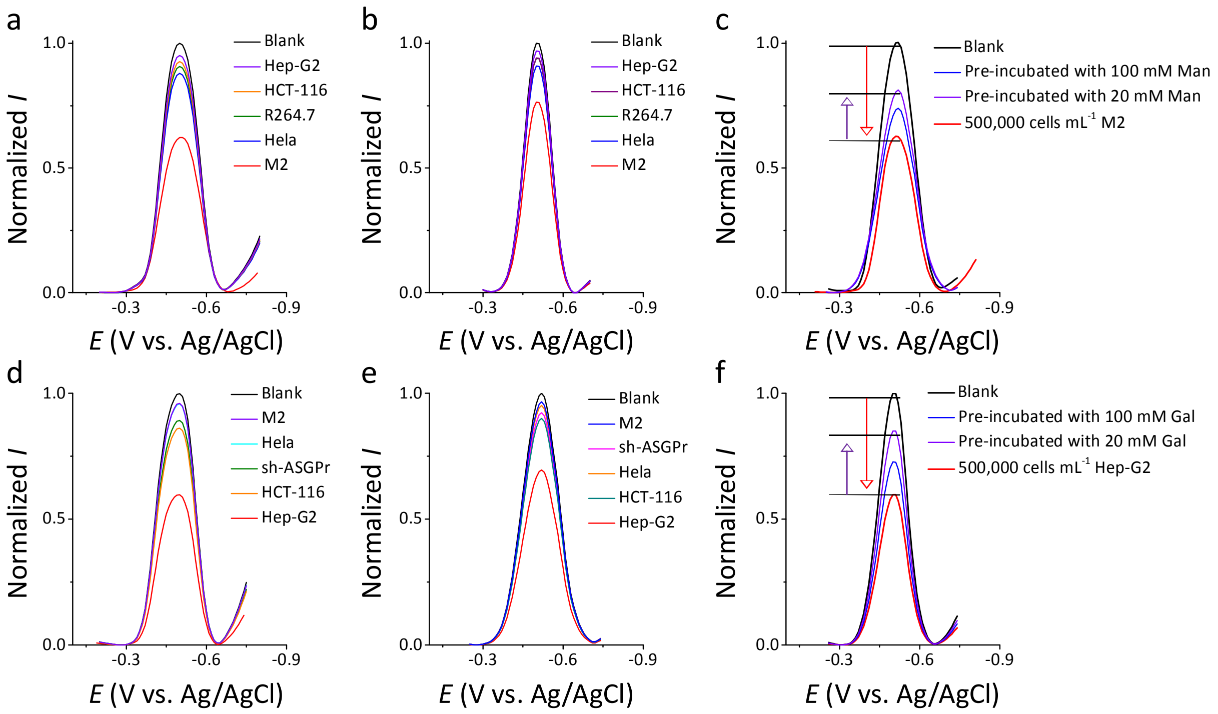

Figure S7. DPVs of (a) **ZBW2@G3** with 500,000 cells mL^{-1} different types of cells, (b) **ZBW2@G3** with 100,000 cells mL^{-1} different types of cells, (c) **ZBW3@G3** with 500,000 cells mL^{-1} different types of cells, (d) **ZBW3@G3** with 100,000 cells mL^{-1} different types of cells, (e) **ZBW2@G3** with 500,000 cells mL^{-1} M2 in the absence or presence of free mannose, and (f) **ZBW2@G3** with 500,000 cells mL^{-1} Hep-G2 in the absence or presence of free galactose.

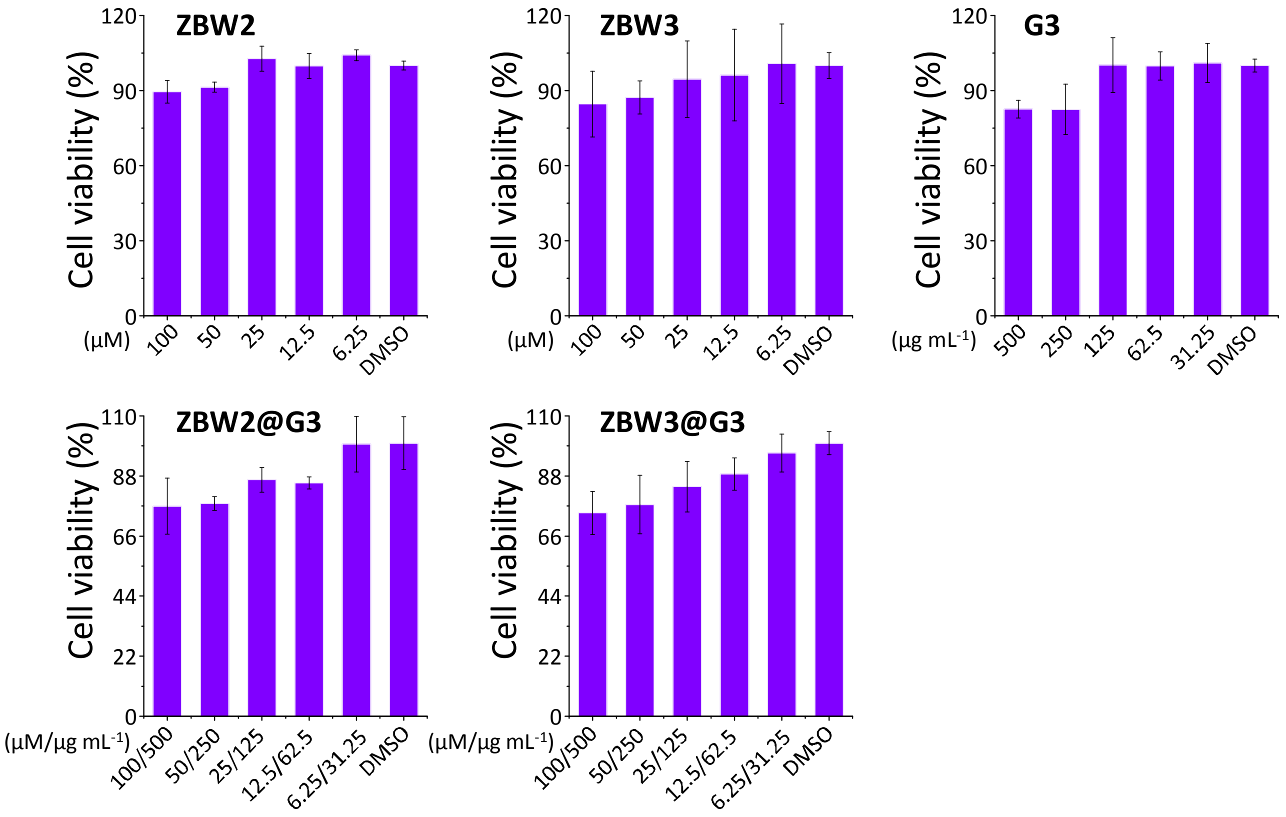

Figure S8. Cytotoxicity of **ZBW2**, **ZBW3**, **G3** and the composites with increasing concentrations for Hep-G2 cells (measured by CCK-8 assay).

S2. Experimental section (including Scheme S1-S3)

General. All purchased chemicals and reagents are of analytical grade. GOs with different sizes were purchased from Nanjing XFNano Materials Tech. Co., Ltd. and used as is. Solvents were purified by standard procedures. Reactions were monitored by TLC (thin-layer chromatography) using E-Merck aluminum precoated plates of Silica Gel. Concanavalin A (Con A), peanut agglutinin (PNA), wheat germ agglutinin (WGA), Pisum satiwun agglutinin (PSA), bovine serum albumin (BSA), and pepsin (Pep) were purchased from Sigma-Aldrich or Shanghai Shrek biotechnology Co., Ltd. The solutions for analytical studies were prepared with demonized water obtained with a Milli-Q System (Billerica, MA, USA). The screen printed electrodes were produced on an AT-25P machine (ATMACHAMP ENT. Corp., Taiwan) using polyester screens with appropriate stencil designs. ^{1}H and ^{13}C NMR spectra were recorded on a Bruker AV-400 or AM-400 spectrometer in $CDCl_3$, DMSO-d_6 or CD_3OD solutions using tetramethylsilane (TMS) as an internal standard. HPLC was carried out on an Agilent 1100 HPLC system. High resolution mass spectra (HRMS) were recorded on a Waters LCT Premier XE spectrometer instrument using standard conditions (ESI, 70 eV).

Differential pulse voltammetry (DPV). Screen-printed electrodes (SPEs) were pretreated in PBS (0.05 M, pH 7.0) containing 0.1 M KCl by applying an anodic potential of 2 V (vs. Ag/AgCl) for 200 s, and were then washed with water three times. The circular area (2 mm in diameter) was used as the working electrode; the reference electrode was printed with 40% AgCl in silver paste, and the auxiliary electrode printed with carbon ink. DPVs were recorded with a computer controlled CHI 1211B electrochemical station (Chenhua Co. Ltd, Shanghai, China) recorded with an amplitude of 0.05 V, a pulse width of 0.2 s, a standing time of 2 s, and a scanning range from -0.8 V to -0.2 V in Tris-HCl (0.01 M, pH 7.3) containing 0.1 M KCl. For electrode functionalization, a drop (4 μL) of compound solution (dissolved in 80% deionized water and 20% CH_3OH) of different concentrations was dripped onto the graphene functionalized working electrode area of SPE and incubated for 30 min. Then the electrodes were rinsed with the buffer solution three times, dried at room temperature, and then immersed in degassed buffer for measurement. For detection of the sugar-lectin interactions, a drop (0.01 M Tris-HCl, containing 0.1 mM Ca^{2+} and Mn^{2+}, 4 μL) of lectin solution was dripped onto the composite electrodes, and the measurement was conducted as indicated above.

Electrochemical impedance spectroscopy (EIS). EIS was performed with a ZAHNER apparatus in the presence of the $[Fe(CN)_6]^{3-}/[Fe(CN)_6]^{4-}$ (5 mM) redox couple in 0.1 M KCl solution in the frequency range of 10 mHz to 100 KHz (perturbation signal: 5 mV). All data collected were fitted with the software ZSimpWin.

Atomic force microscopy (AFM). AFM were performed with a NanoScope IIIa controller (Veeco, USA) operating in the tapping mode. The samples used were prepared by depositing the corresponding dispersion on the mica sheet, and then dried under vacuum at room temperature.

Fourier Transform Infrared Spectroscopy (FTIR). FTIR spectra were recorded on a Nicolet 380 FTIR spectrometer (Thermo Electron Corporation, USA). The samples were mixed with KBr and then compressed into pellets for analysis in the spectral range of $\tilde{\nu}$ = 4000 to 500 cm^{-1}. All baselines of the spectra were corrected.

Raman Spectroscopy. Raman spectra were performed on a Renishaw InVia Reflex Raman system (Renishaw plc, Wotton-under-Edge, UK) that employs a grating spectrometer with a Peltier-cooled charge-coupled device (CCD) detector coupled to a confocal microscope, which were then processed with Renishaw WiRE 3.2 software. The Raman scattering was excited by an argon ion laser (*l* = 514.5 nm).

Cell culture. Cells were obtained from ATCC (Rockville, MD), which were cultured in a Dulbecco's Modified Eagle's Medium (Invitrogen, Carlsbad, CA, USA) supplemented with a 10% Fetal bovine serum (Gibco, Gland Island, NY, USA) at 37 °C in a 5% humidified CO_2 air environment.

Silencing of ASGP-R1 gene by RNA interference. Silencing of the ASGP-R1 gene expression on Hep-G2 cells was mediated by an ASGPR-1 gene-specific siRNA oligonucleotide duplex purchased from Santa Cruz Biotechnologies (Santa Cruz, CA). Briefly, HepG2 cells were seeded at 400,000 cells/well in a 6-well plate, and lipofectamine 2000 (Invitrogen) was used for transfection as instructed by the manufacturer. After treating the cells with siRNAs for 6 h, the lipofetamine-siRNA

complex containing medium was replaced. After another 48 h, the cells were harvested for experiments and the level of ASGP-R1 mRNA was evaluated by quantitative real-time PCR.

Macrophage culture. RAW264.7 macrophage cells were purchased from Shanghai Cell Bank, Chinese Academy of Sciences. The cells were grown in 1640-RMPI (GIBCO) supplemented with 10% FBS (GIBCO), in a humidified atmosphere at 37 °C with 5% CO_2. To induce M2 macrophages, the cells were treated with IL-4 (20 ng/mL) in the growth medium for 24 hours.

CCK-8 cytotoxicity assay. Cell viability was measured using a cell counting kit-8 (CCK-8, Dojindo, Rockville, MD). 1×10^4 cells were seeded in a 96-well plate and cultured in HG-DMEM supplemented with 10% FBS and 1% PS at 37 °C under 5% CO_2. After 10 h, the cells were washed with 100 μL of serum-free HG-DMEM (1% PS) two times and incubated with 100 μL of different concentrations of compounds and composites in serum-free HG-DMEM (1% PS). After exposure for 24 h, the cells were washed twice with serum-free HGDMEM and 10 μL of CCK-8 solution was added to each well containing 100 μL of serum-free HGDMEM, followed by a gentle shake. After incubation for 2 h at 37 °C under 5% CO_2, 80 μL of the mixture was transferred to another 96-well plate. The absorbance of the mixture solutions was measured at 450 nm using an M5 microplate reader (Molecular Device, USA).

Quantitative RT-qPCR. Total RNA was isolated from the cells using TRIzol according to the manufacturer (TaKaRa)). Total RNA (1 μg) were reverse-transcripted using the Prime Script RT reagent kit (TaKaRa). The RT-PCR was performed using the strata gene MX3005P Detection system (Agilent technologies) for quantification with SYBR Green and melting curve analysis. Gene expression was normalized to GAPDH or actin expression and assessed using the $\Delta(\Delta Ct)$ calculation method. Primer sequences were as follows:

Mouse-MRC1 forward primer: GGAGTGATGGAACCCCAGTG

Mouse-MRC1 reverse primer: ACCCTCCGGTACTACAGCAT

Human MRC1 forward primer: ACTGAATTGTACTGGTCTGTCCT

Human MRC1 reverse primer: GCTGACATCAGCTACCCATCA

Human ASGPR-1 forward primer: CTGGACAATGAGGAGAGTGAC

Human ASGPR-1 reverse primer: TTGAAGCCCGTCTCGTAGTC

Mouse ASGPR-1 forward primer: CTCCTTAGCCTGGGCTCTTC

Mouse ASGPR-1 reverse primer: CCGGAGTTGGGAATTTTGGGA

Human GAPDH forward primer: ATCACTGCCACCCAGAAGAC

Human GAPDH reverse primer: ATGAGGTCCACCACCCTGTT

Mouse GAPDH forward primer: GGTTGTCTCCTGCGACTTCA

Mouse GAPDH reverse primer: TAGGGCCTCTCTTGCTCAGT

Synthesis of mono-clicked intermediates. To a soln. of dipropargyl anthraquinone **d** (1.3 equiv.) and azido glycoside (1.0 equiv.) in a solvent mixture of CH_2Cl_2 (5 mL) and H_2O (5 mL) were added $CuSO_4{\cdot}5H_2O$ (2.0 equiv.) and Na ascorbate (4.0 equiv.). This mixture was stirred overnight and then diluted with CH_2Cl_2 and washed with brine. The combined organic layer was dried over $MgSO_4$, filtered, concentrated in vacuum to give a crude product which was then purified by column chromatography. The purified product (1.0 equiv.) was then dissolved in a solvent mixture of MeOH (5 mL) and H_2O (5 mL), followed by addition of excessive Et_3N. This mixture was stirred at room temperature for 36 h. Then, solvent was removed in vacuum, and the residue was purified by column chromatography.

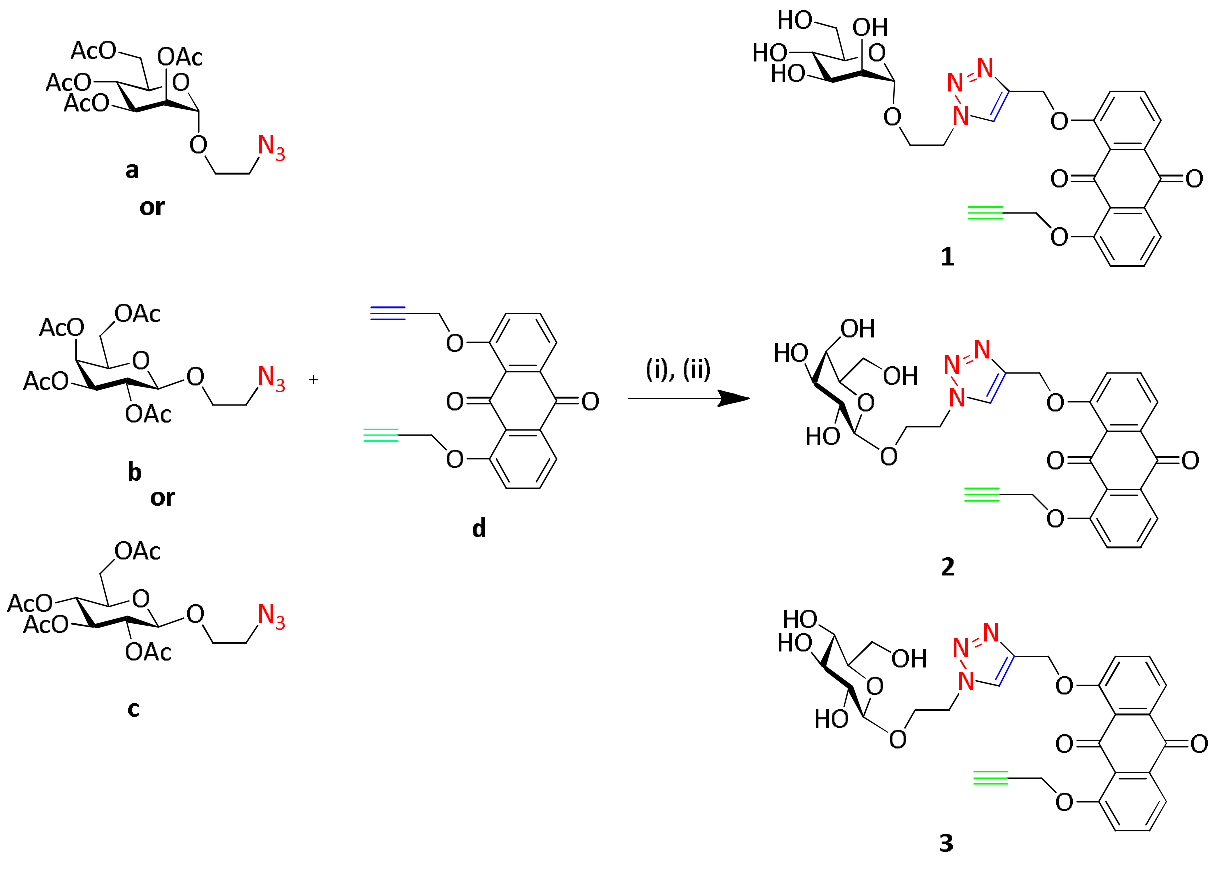

Scheme S1. Reagents and conditions: (i), $CuSO_4{\cdot}5H_2O$/Na ascorbate, CH_2Cl_2/H_2O; (ii), Et_3N, MeOH/H_2O.

*Compound (**1**).* From **a** (560 mg, 1.34 mmol) and **d** (483.5 mg, 1.75 mmol), column chromatography (EtOAc/EtOH = 20:1, v/v) afforded **1** (322.6 mg, 42.6%) as a yellow solid. R_f = 0.15

(EtOAc/EtOH = 10:1, v/v). ^{1}H NMR (400 MHz, CD_3OD): δ 8.24 (s, 1H), 7.76 (dd, J = 7.6, 0.8 Hz, 1H), 7.73 (dd, J = 8.0, 0.8 Hz, 1H), 7.69-7.62 (m, 2H), 7.55 (d, J = 8.4 Hz, 2H), 5.33 (brs, 2H), 4.93 (d, J = 2.4 Hz, 2H), 4.75 (d, J = 1.6 Hz, 1H), 4.70-4.65 (m, 2H), 4.19-4.13 (m, 1H), 3.94-3.89 (m, 1H), 3.80-3.77 (m, 2H), 3.68-3.55 (m, 3H), 3.29-3.24 (m, 1H), 3.07 (t, J = 4.4 Hz, 1H); ^{13}C NMR (100 MHz, CD_3OD): δ 184.5, 184.0, 159.4, 158.5, 144.8, 136.0, 135.9, 135.6,135.3, 126.5, 125.5, 125.3, 121.9, 121.8, 120.8, 120.6, 101.7, 79.2, 78.2, 75.0, 72.5, 71.9, 68.4, 66.8, 64.3, 62.8, 57.9, 51.4; HR-ESI-MS: calcd. for $[C_{28}H_{27}N_3O_{10} + Na]^+$ 588.1594, found 588.1600.

*Compound (**2**).* From **b** (300 mg, 0.72 mmol) and **d** (259.2 mg, 0.94 mmol), column chromatography (EtOAc/EtOH = 20:1, v/v) afforded **2** (197.4 mg, 48.5%) as a yellow solid. R_f = 0.30 (EtOAc/EtOH = 10:1, v/v). ^{1}H NMR (400 MHz, CD_3OD): δ 8.38 (s, 1H), 7.83 (t, J = 8.4 Hz, 2H), 7.73 (t, J = 7.2 Hz, 2H), 7.66-7.60 (m, 2H), 5.38 (s, 2H), 4.98 (s, 2H), 4.72 (t, J = 4.0 Hz, 2H), 4.31 (d, J = 7.2 Hz, 2H), 4.04-4.10 (m, 1H), 3.83 (brs, 1H), 3.77 (d, J = 6.8 Hz, 1H), 3.72 (dd, J = 11.2, 4.8 Hz, 1H), 3.53-3.58 (m, 2H), 3.45 (d, J = 10 Hz, 1H), 3.09 (s, 1H); ^{13}C NMR (100 MHz, CD_3OD): δ 164.8, 159.2, 158.4, 144.2, 135.8, 135.4, 135.1, 130.9, 128.8, 126.9, 121.7, 120.6, 120.4, 105.1, 79.1, 78.0, 76.7, 74.7, 72.2, 70.2, 68.9, 64.0, 62.4, 59.6, 57.7, 52.5. HR-ESI-MS: calcd. for $[C_{28}H_{27}N_3O_{10} + Na]^+$ 588.1594, found 588.1599.

*Compound (**3**).* From **c** (650 mg, 1.56 mmol) and **d** (640 mg, 2.02 mmol), column chromatography (EtOAc/EtOH = 30:1, v/v) afforded **3** (480.4 mg, 54.5%) as a yellow solid. R_f = 0.30 (EtOAc/EtOH =10:1, v/v). ^{1}H NMR (400 MHz, CD_3OD): δ 8.24 (s, 1H), 7.72 (t, J = 8.0 Hz, 2H), 7.64-7.59 (m, 2H), 7.54 (d, J = 1.2 Hz, 1H), 7.50 (dd, J = 9.2, 0.8 Hz, 1H), 5.27 (s, 2H), 4.86 (d, J = 2.4 Hz, 2H), 4.60 (t, J = 5.6 Hz, 2H), 4.23 (d, J = 8.0 Hz, 1H), 4.20-4.15 (m, 1H), 3.98 (s, 1H), 3.96-3.92 (m, 1H), 3.78-3.76 (m, 1H), 3.57-3.50 (m, 2H), 3.18-3.15 (m, 2H), 2.96 (t, J = 2.4 Hz, 1H); ^{13}C NMR (100 MHz, CD_3OD): δ 184.6, 159.4, 158.6, 136.1, 136.0, 135.6, 135.3, 127.0, 125.5, 121.9, 120.8, 120.6, 104.7, 79.2, 78.2, 78.1, 78.0, 75.0, 72.7, 71.5, 64.2, 62.7, 57.8, 53.6, 53.1. HR-ESI-MS: calcd. for $[C_{28}H_{27}N_3O_{10} + Na]^+$ 588.1594, found 588.1597.

Synthesis of dual-clicked pyrenyl glycoanthraquinones. To a soln. of mono-propargyl (1.0 equiv.) and **g** (1.2 equiv.) in a solvent mixture of CH_2Cl_2 (3 mL), tert butyl alcohol (TBA, 5mL) and H_2O (5 mL) were added $CuSO_4{\cdot}5H_2O$ (2.0 equiv.) and Na ascorbate (4.0 equiv.). This mixture was stirred at reflux (60 ^{o}C) over night, concentrated in vacuum to remove TBA, and then diluted with CH_2Cl_2 and washed with brine. The combined organic layer was dried over $MgSO_4$, filtered, concentrated in vacuum to give a crude product which was then purified by column chromatography.

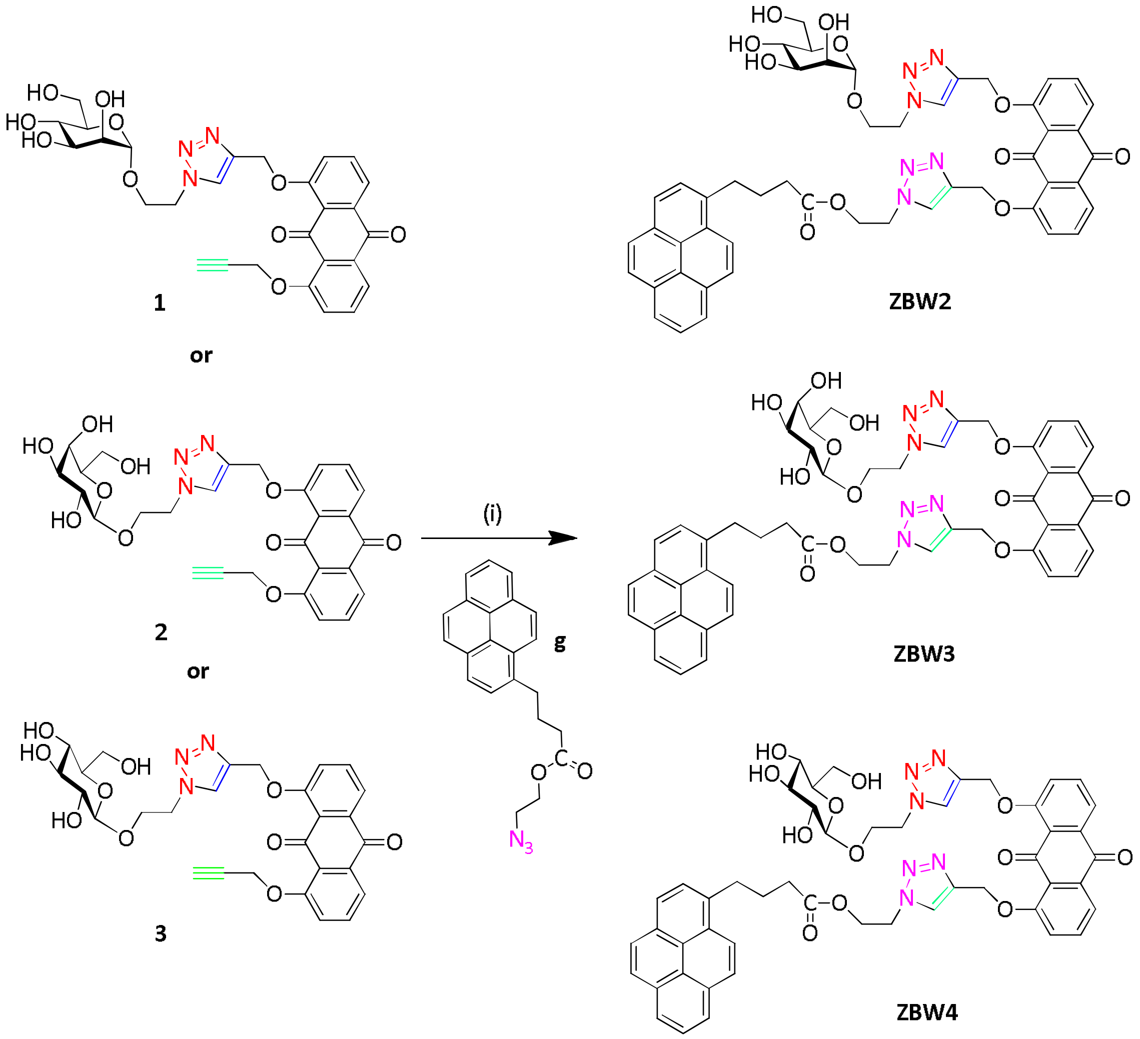

Scheme S2. Reagents and conditions: (i) $CuSO_4{\cdot}5H_2O$/Na ascorbate, CH_2Cl_2/H_2O/TBA.

ZBW2. From **1** (160 mg, 0.29 mmol) and **g** (121.4 mg, 0.34 mmol), column chromatography (EtOAc/EtOH = 5:1, v/v) afforded **ZBW2** (94.7 mg, 36%) as a yellow solid. R_f = 0.45 (EtOAc/EtOH = 1:1, v/v). ^{1}H NMR (400 MHz, CD_3OD): δ 8.26 (s, 1H), 8.18-8.11 (m, 1H), 7.95 (d, J = 7.6 Hz, 1H), 7.83

(t, *J* = 7.6 Hz, 1H), 7.74-7.67 (m, 3H), 7.66-7.58 (m, 2H), 7.46-7.44 (m, 2H), 7.30 (t, *J* = 6.8 Hz, 1H), 7.22 (dd, *J* = 8.4, 2.4 Hz, 2H), 5.43 (s, 2H), 5.35-5.33 (m, 1H), 4.74-4.69 (m, 3H), 4.61 (brs, 2H), 4.44 (s, 1H), 4.21 (dd, *J* = 6.0, 2.0 Hz, 2H), 4.15-4.10 (m, 2H), 3.79-3.75 (m, 3H), 3.66-3.61 (m, 3H), 3.59-3.56 (m, 3H), 3.48 (s, 1H), 3.13 (s, 1H), 2.76-2.72 (m, 1H), 2.46-2.42 (m, 1H), 2.37-2.34 (m, 2H); ^{13}C NMR (100 MHz, CD_3OD): δ 195.2, 195.0, 194.5, 189.8, 184.6, 184.1, 158.5, 136.0, 135.6, 135.3, 130.9, 126.6, 125.5, 121.9, 121.8, 120.9, 120.7, 107.2, 103.9, 101.7, 101.4, 79.2, 78.2, 75.0, 72.5, 71.9, 68.3, 66.8, 64.2, 62.8, 57.8, 51.4, 33.1, 23.8. HR-ESI-MS: calcd. for $[C_{50}H_{46}N_6O_{12} + Na]^+$ 945.3071, found 945.3077. HPLC: t_R = 5.04 min over 15 min of eluent (methanol/H_2O = 9:1, v/v), purity 91.1%.

ZBW3. From **2** (190 mg, 0.43 mmol) and **g** (182.9 mg, 0.51 mmol), column chromatography (EtOAc/EtOH = 5:1, v/v) afforded **ZBW3** (126.8 mg, 32%) as a yellow solid. R_f = 0.35 (EtOAc/EtOH = 1:1, v/v). ^{1}H NMR (400 MHz, CD_3OD): δ 8.11 (s, 1H), 7.93 (d, *J* = 8.0 Hz, 1H), 7.82 (t, *J* = 8.0 Hz, 2H), 7.75-7.70 (m, 3H), 7.66-7.64 (m, 2H), 7.59 (d, *J* = 8.4 Hz, 1H), 7.46-7.44 (m, 1H), 7.29 (dd, *J* = 15.2, 8.0 Hz, 2H), 7.22 (dd, *J* = 8.8, 2.8 Hz, 1H), 5.41 (s, 1H), 5.34 (d, *J* = 8.4 Hz, 2H), 4.72-4.70 (m, 2H), 4.64 (s, 3H), 4.47-4.38 (m, 2H), 4.30-4.26 (m, 3H), 4.07-4.03 (m, 2H), 3.91 (s, 1H), 3.83 (d, *J* = 2.8 Hz, 2H), 3.75-3.71 (m, 3H), 3.51 (d, *J* = 7.2 Hz, 3H), 3.48 (d, *J* = 3.2 Hz, 2H), 2.37 (s, 1H), 2.34 (s, 1H); ^{13}C NMR (100 MHz, DMSO-d_6): δ 172.2, 165.9, 164.5, 147.2, 140.3, 137.6, 136.9, 136.8, 135.9, 134.1, 133.9, 129.6, 123.5, 118.3, 108.8, 102.8, 96.9, 88.7, 85.8, 85.3, 81.9, 80.6, 78.6, 75.7, 75.6, 74.3, 73.4, 72.6, 65.7, 64.7, 63.8, 36.5, 31.8. HR-ESI-MS: calcd. for $[C_{50}H_{46}N_6O_{12} + Na]^+$ 945.3071, found 945.3082. HPLC: t_R = 4.25 min over 15 min of eluent (methanol/H_2O = 9:1, v/v), purity 98.9%.

ZBW4. From **3** (300 mg, 0.54 mmol) and **g** (227.4 mg, 0.64 mmol), column chromatography (EtOAc/EtOH = 5:1, v/v) afforded **ZBW4** (163.5 mg, 33.8%) as a yellow solid. R_f = 0.35 (EtOAc/EtOH = 1:1, v/v). ^{1}H NMR (400 MHz, CD_3OD): δ 8.15 (s, 2H), 8.09 (d, *J* = 8.0 Hz, 1H), 7.74 (dd, *J* = 8.8, 1.2 Hz, 3H), 7.71 (d, *J* = 1.2 Hz, 1H), 7.65 (d, *J* = 1.6 Hz, 1H), 7.63 (d, *J* = 1.6 Hz, 2H), 7.60 (t, *J* = 2.0 Hz, 2H), 7.54 (d, *J* = 1.2 Hz, 1H), 7.49 (d, *J* = 1.2 Hz, 1H), 5.24 (t, *J* = 4.8 Hz, 2H), 5.03 (s, 1H), 4.72-4.69 (m, 2H), 4.64 (d, *J* = 0.8 Hz, 2H), 4.63 (dd, *J* = 5.2, 1.6 Hz, 1H), 4.60 (s, 1H), 4.60-4.56 (m, 4H), 4.08-4.03 (m, 3H), 3.84-3.78 (m, 3H), 3.70 (d, *J* = 2.0 Hz, 1H), 3.67-3.66 (m, 3H), 3.17 (d, *J* = 2.0 Hz, 1H), 3.16-3.15 (m, 1H), 3.13 (d, *J* = 2.0 Hz, 1H), 2.97 (t, *J* = 2.4 Hz, 2H); ^{13}C NMR(100 MHz, CD_3OD): δ 165.0, 156.4,

154.0, 151.1, 145.8, 142.4, 141.2, 133.4, 131.5, 130.1, 128.4, 127.3, 117.6, 107.8, 107.4, 107.3, 80.7, 78.5, 78.0, 77.6, 77.5, 77.4, 76.8, 74.6, 74.2, 70.0, 68.8, 58.1. HR-ESI-MS: calcd. for $[C_{50}H_{46}N_6O_{12} + Na]^+$ 945.3071, found 945.3072. HPLC: t_R = 5.63 min over 15 min of eluent (methanol/H_2O = 9:1, v/v), purity 95.3%.

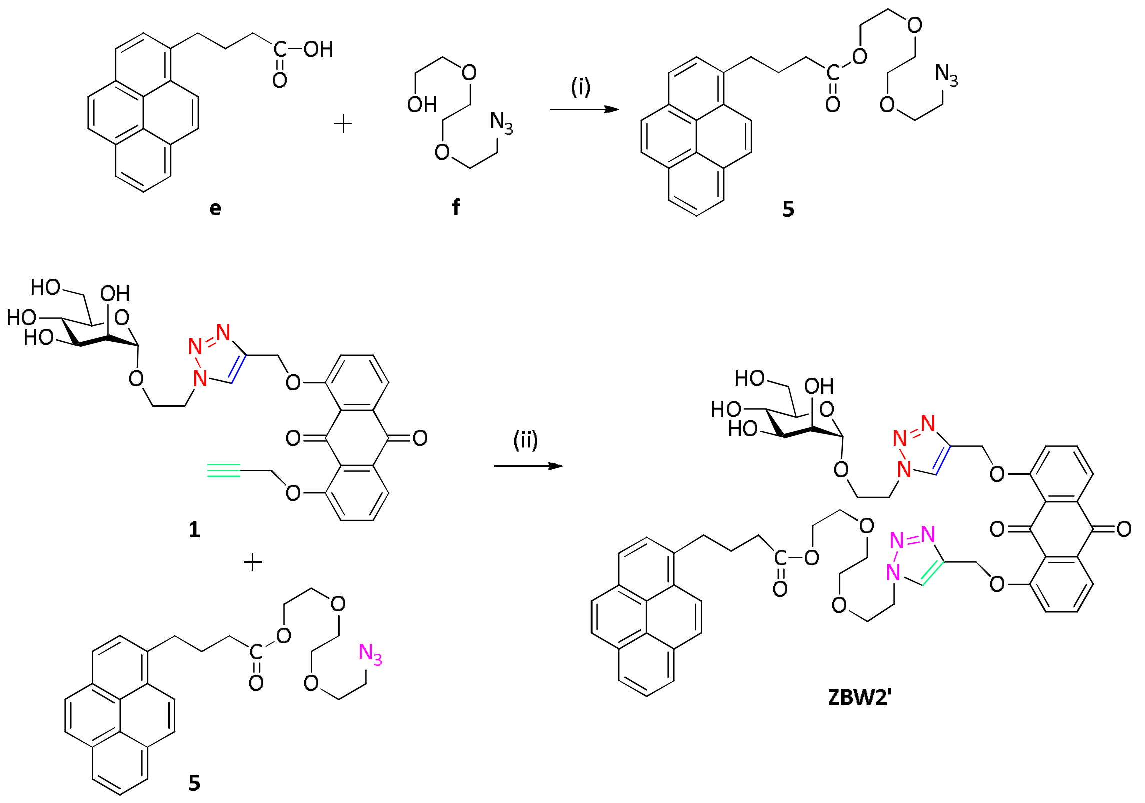

Scheme S3 . Reagents and conditions: (i) EDC·HCl/DMAP, DCM; (ii) Reagents and conditions: $CuSO_4 \cdot 5H_2O$/Na ascorbate, CH_2Cl_2/H_2O/TBA.

*Synthesis of **5***: To a soln. of pyrene-1-butyric acid **e** (500 mg, 1.74 mmol) and **f** (456 mg, 2.60 mmol) in CH_2Cl_2 (5 mL) were added EDC·HCl (1.2 equiv.) and DMAP (0.5 equiv.). This mixture was stirred at room temperature for 5 h and then diluted with CH_2Cl_2 and washed with brine. The combined organic layer was dried over $MgSO_4$, filtered, concentrated in vacuum to give a crude product which was then purified by column chromatography (petroleum ether [PE]/EtOAc = 40:1) afforded **5** (565.2 mg, 73%) as a white solid. R_f = 0.75 (PE/EtOAc = 10:1). ^{1}H NMR (400 MHz, $CDCl_3$): δ 8.31 (d, J = 9.2 Hz, 1H), 8.18-8.16 (m, 2H), 8.13 (d, J = 2.4 Hz, 1H), 8.11 (s, 1H), 8.03 (s, 2H), 7.99 (t, J = 7.6 Hz,

1H), 7.87 (d, J = 8.0 Hz, 1H), 4.26 (t, J = 4.8 Hz, 2H), 3.73-3.67 (m, 4H), 3.63 (d, J = 2.8, 2H), 3.60 (t, J = 4.8 Hz, 2H), 3.40 (t, J = 7.6 Hz, 2H), 3.31 (t, J = 4.8 Hz, 2H), 2.50 (t, J = 7.2 Hz, 2H), 2.24-2.17 (m, 2H). LR-ESI-MS: calcd. for $[C_{26}H_{27}N_3O_4 + Na]^+$ 468.19, found 468.22.

*Synthesis of **ZBW2'***: To a soln. of the **1** (150 mg, 0.27 mmol) and **5** (142 mg, 0.32 mmol) in a solvent mixture of CH_2Cl_2 (3 mL), TBA (5 mL) and H_2O (5 mL) were added $CuSO_4{\cdot}5H_2O$ (2.0 equiv.) and Na ascorbate (4.0 equiv.). This mixture was refluxed at 60 ^{o}C overnight, concentrated in vacuum to remove TBA, and then diluted with CH_2Cl_2 and washed with brine. The combined organic layer was dried over $MgSO_4$, filtered, concentrated in vacuum to give a crude product which was then purified by column chromatography (EtOAc/EtOH = 5:1) to afford **ZBW2'** (103.5 mg, 38%) as a yellow solid. R_f = 0.40 (EtOAc/EtOH = 1:1). ^{1}H NMR (400 MHz, CD_3OD): δ 8.18 (s, 1H), 7.81 (d, J = 7.6 Hz, 2H), 7.72 (t, J = 8.0 Hz, 2H), 7.61-7.54 (m, 4H), 7.36-7.34 (m, 1H), 7.19-7.11 (m, 2H), 5.32 (s, 3H), 5.24 (t, J = 4.8 Hz, 2H), 4.68 (d, J = 1.2 Hz, 2H), 4.61 (dd, J = 9.6, 5.6 Hz, 3H), 4.52 (brs, 3H), 4.09-4.04 (m, 2H), 3.86-3.81 (m, 2H), 3.72-3.68 (m, 3H), 3.65-3.64 (d, J = 2.0 Hz, 1H), 3.59 (d, J = 5.6 Hz, 1H), 3.56 (d, J = 5.2 Hz, 1H), 3.54-3.50 (m, 4H), 3.25 (s, 1H), 3.16-3.11 (m, 2H), 2.36-2.34 (m, 1H), 2.16-2.09 (m, 3H), 1.98-1.90 (m, 4H); ^{13}C NMR (100 MHz, CD_3OD): δ 183.7, 163.4, 160.7, 137.1, 137.0, 134.1, 132.3, 130.9, 125.5, 124.2, 122.4, 121.9, 121.5, 119.6, 118.2, 101.7, 75.0, 74.9, 72.5, 71.9, 68.4, 66.8, 64.4, 62.8, 61.6, 51.4, 51.3, 49.9. HR-ESI-MS: calcd. for $[C_{54}H_{54}N_6O_{14} + Na]^+$ 1033.3596, found 1033.3595. HPLC: t_R = 3.92 min over 12 min of eluent (methanol/H_2O = 9:1, v/v), purity 92.5%.

S3. NMR copies of new compounds

^{1}H NMR of **1**:

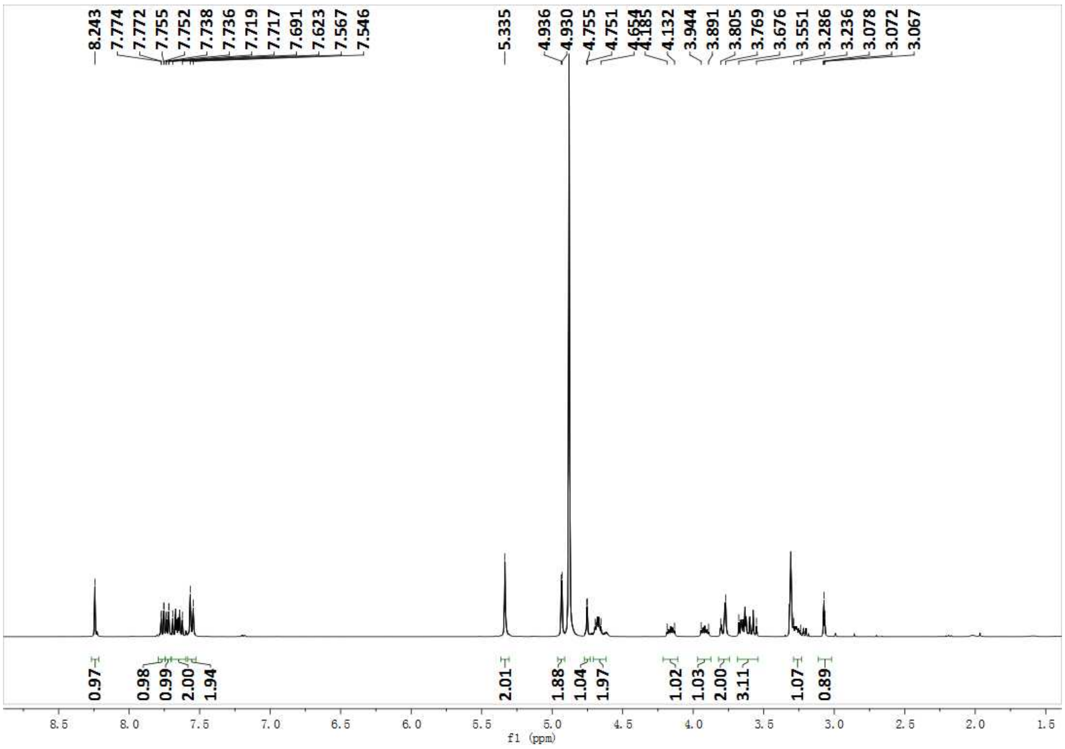

^{13}C NMR of **1**:

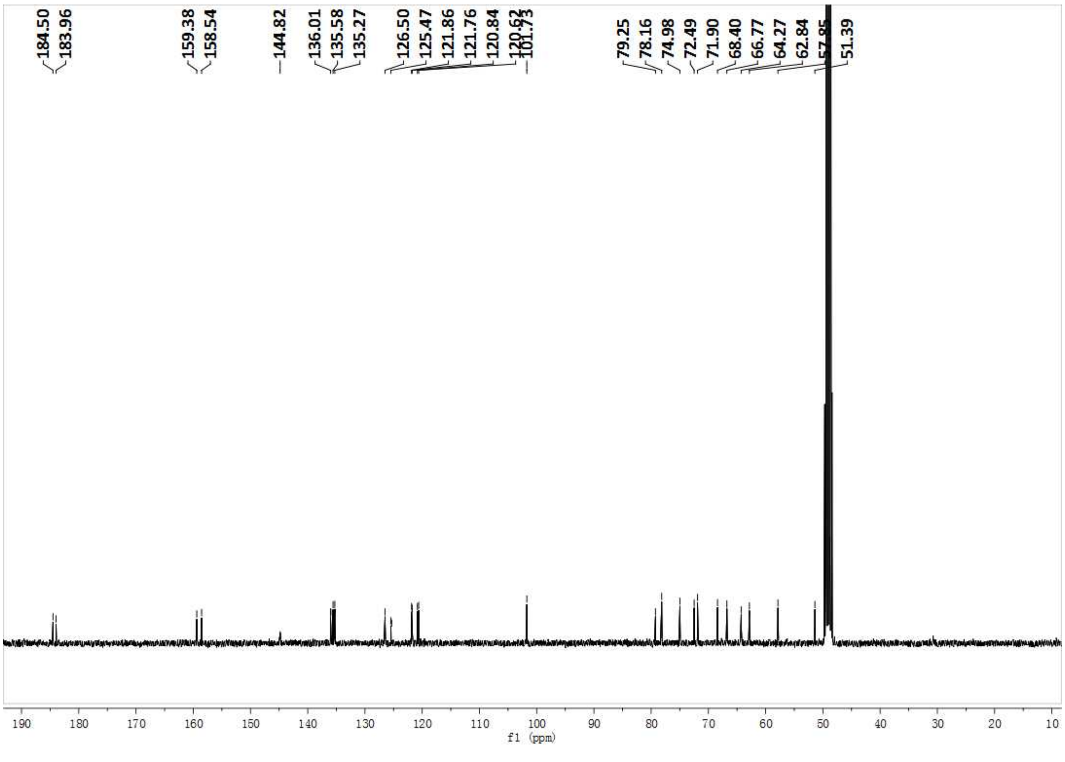

^{1}H NMR of **2**:

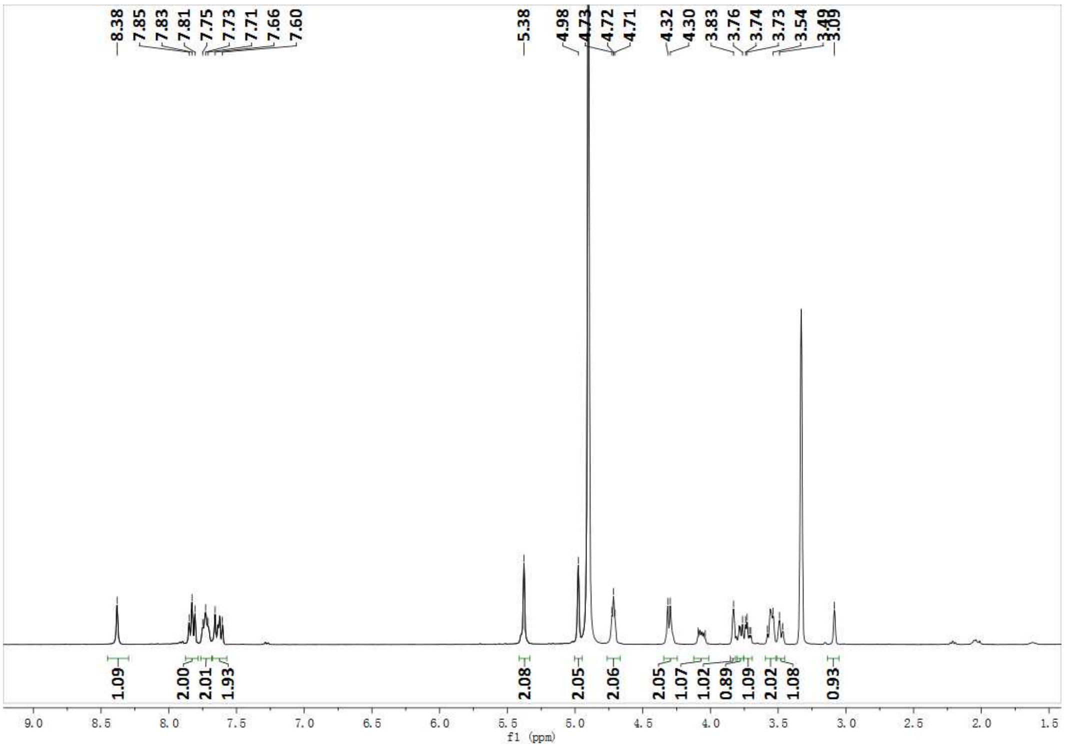

^{13}C NMR of **2**:

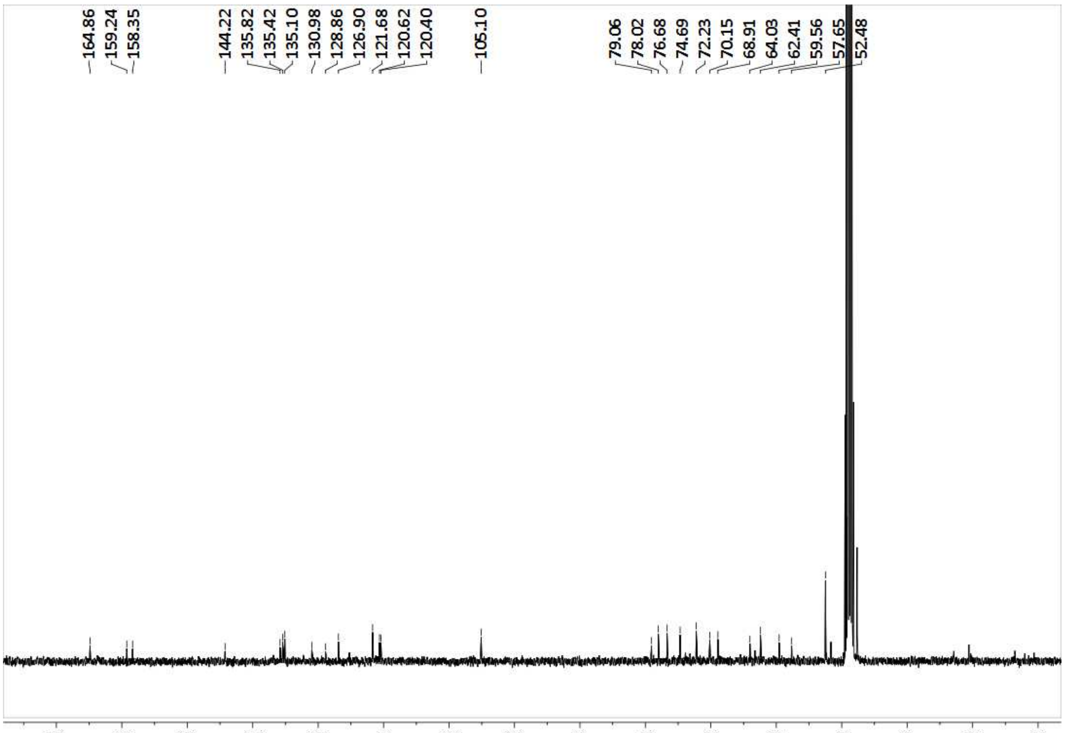

^{1}H NMR of **3**:

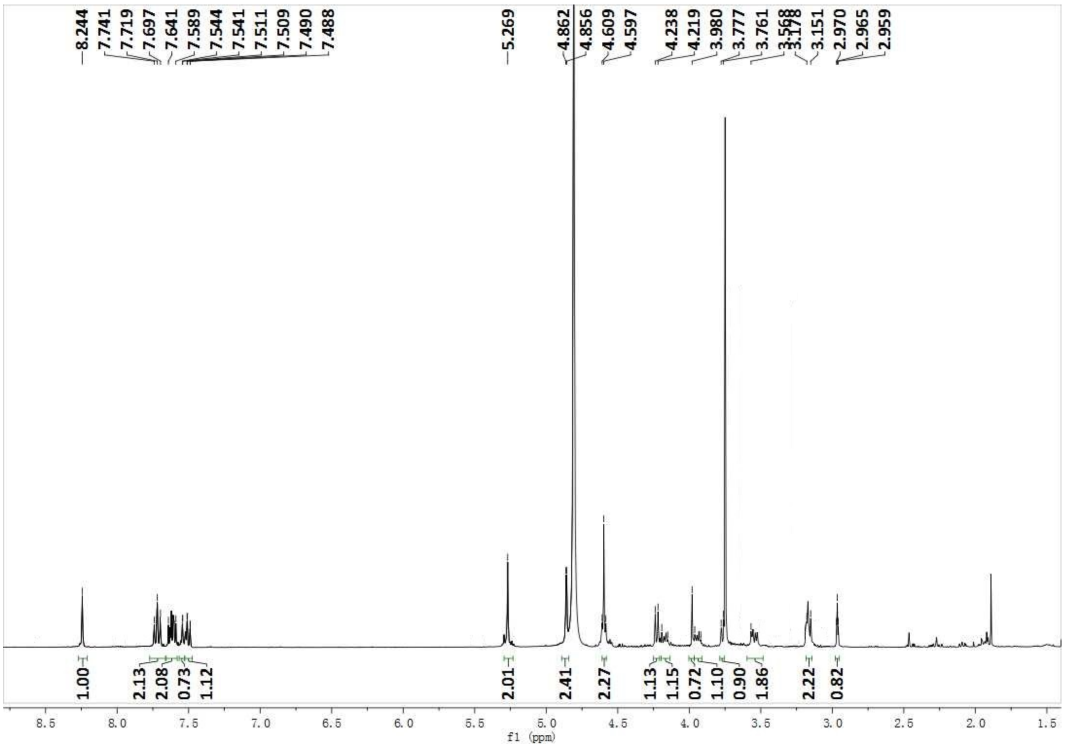

^{13}C NMR of **3**:

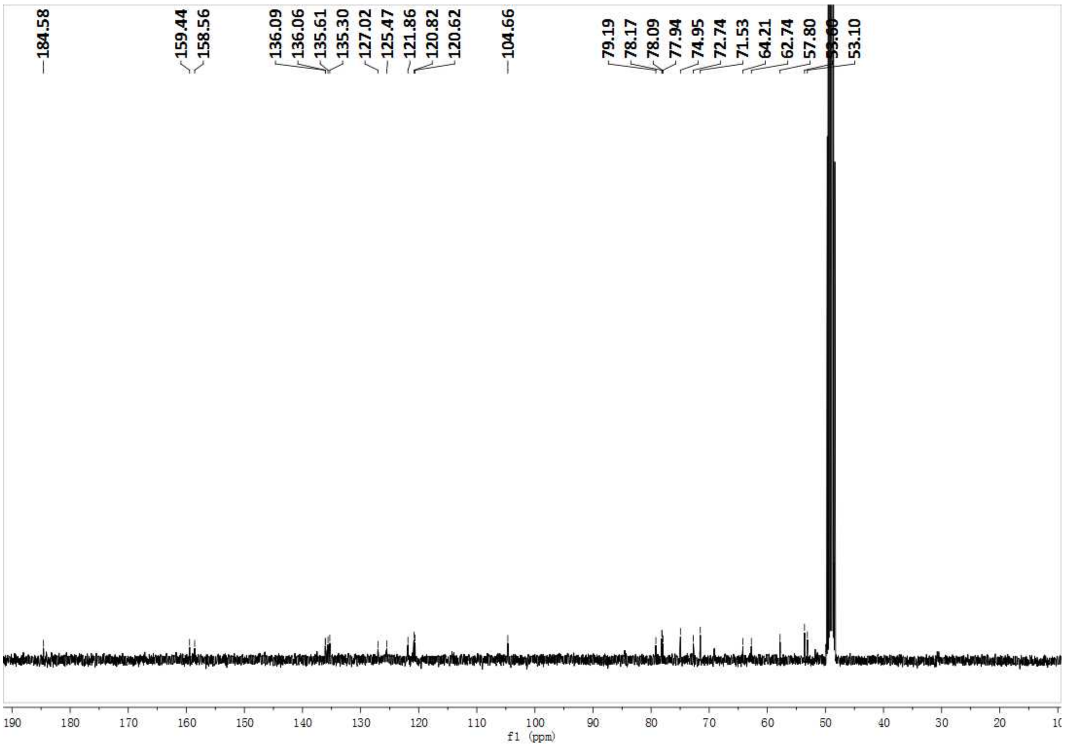

^{1}H NMR of **ZBW2**:

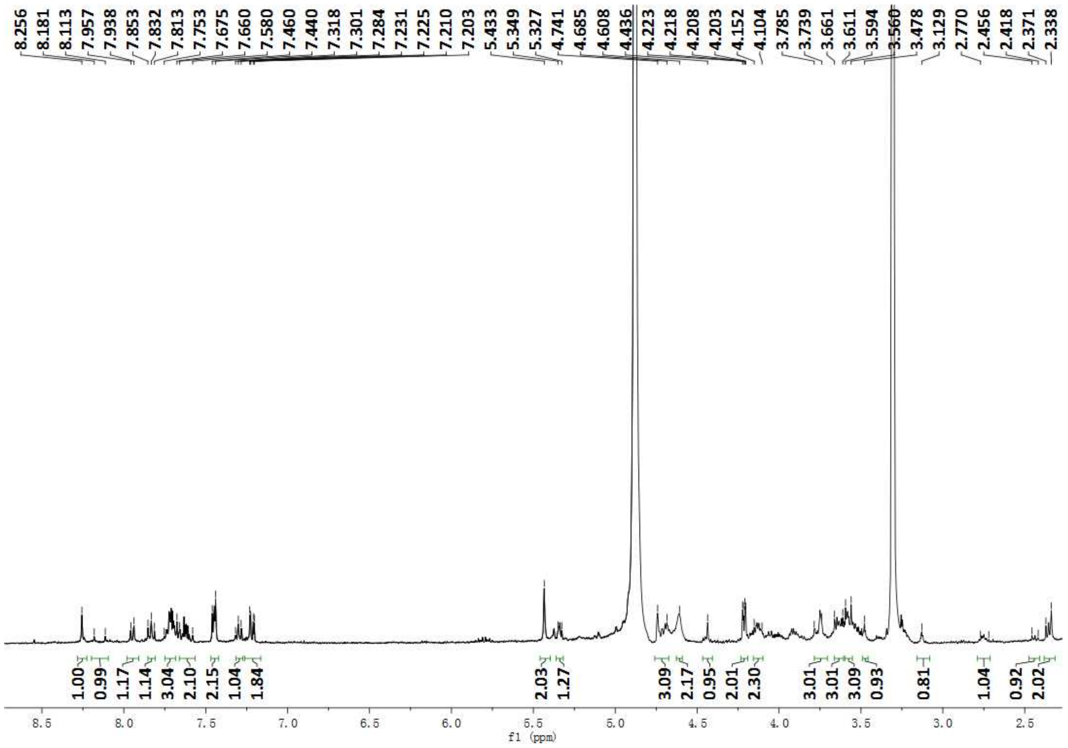

^{13}C NMR of **ZBW2**:

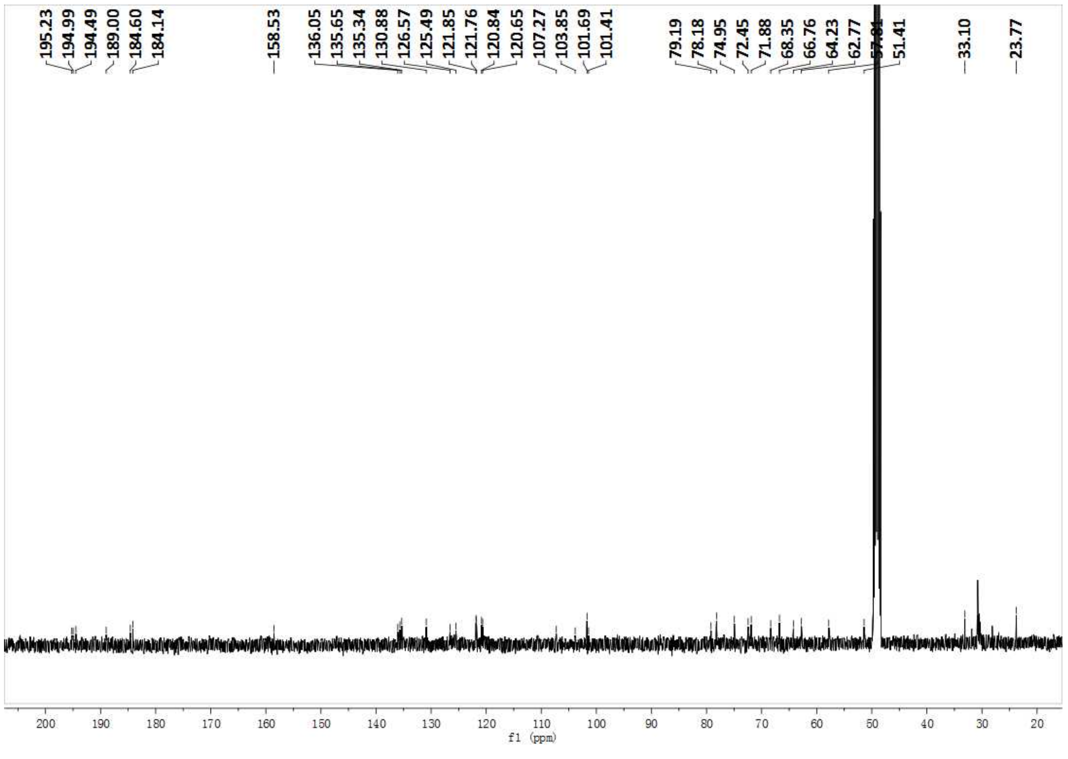

^{1}H NMR of **ZBW3**:

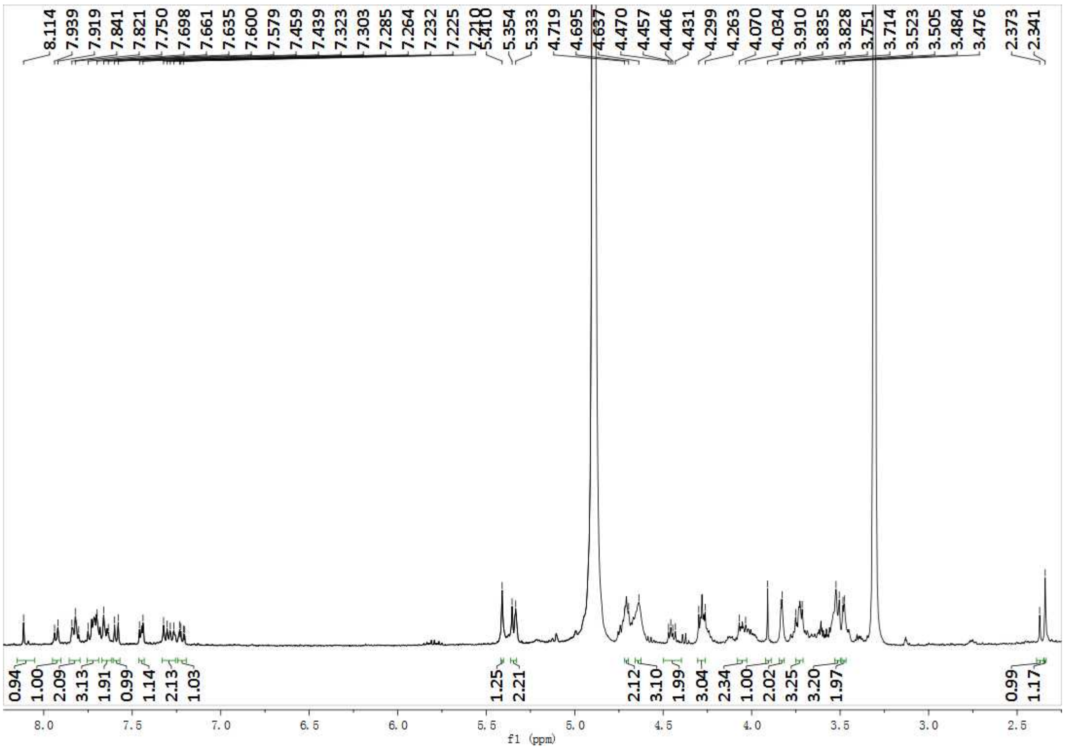

^{13}C NMR of **ZBW3**:

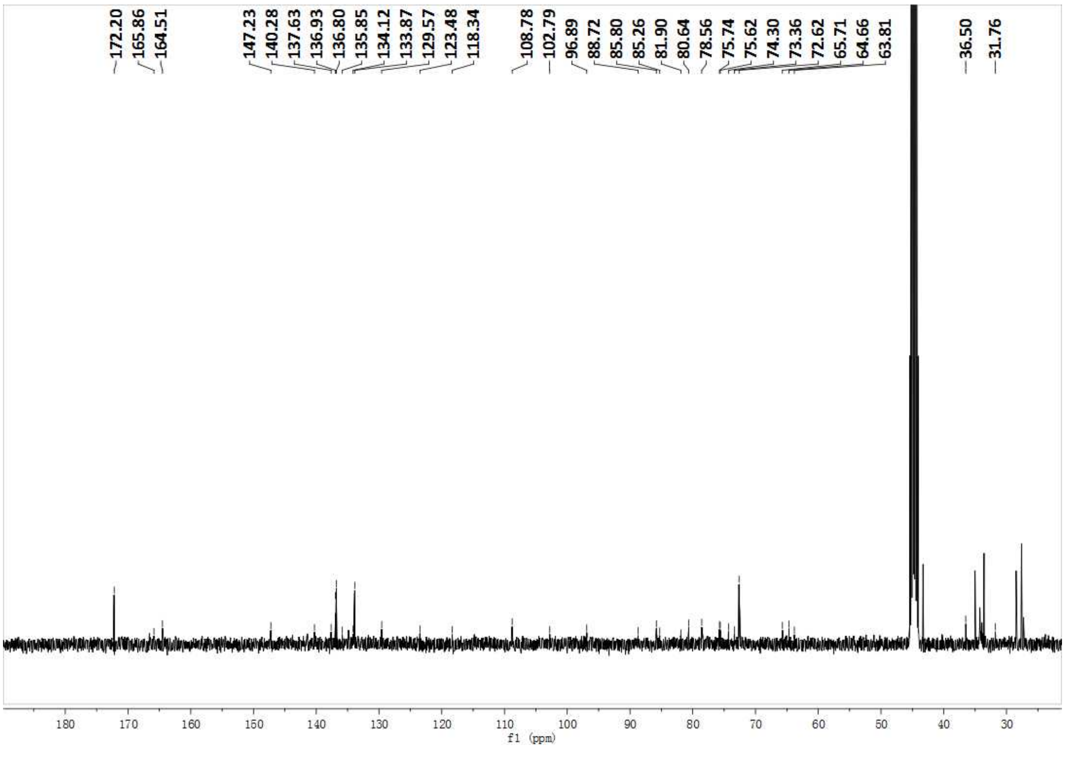

^{1}H NMR of **ZBW4**:

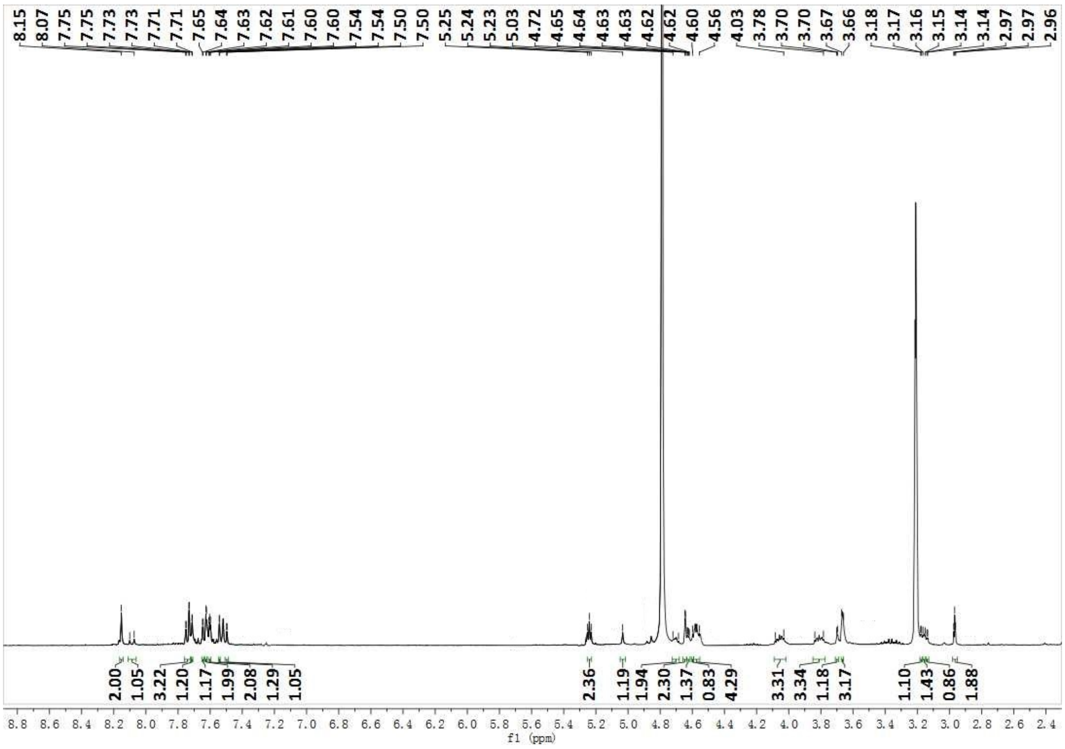

^{13}C NMR of **ZBW4**:

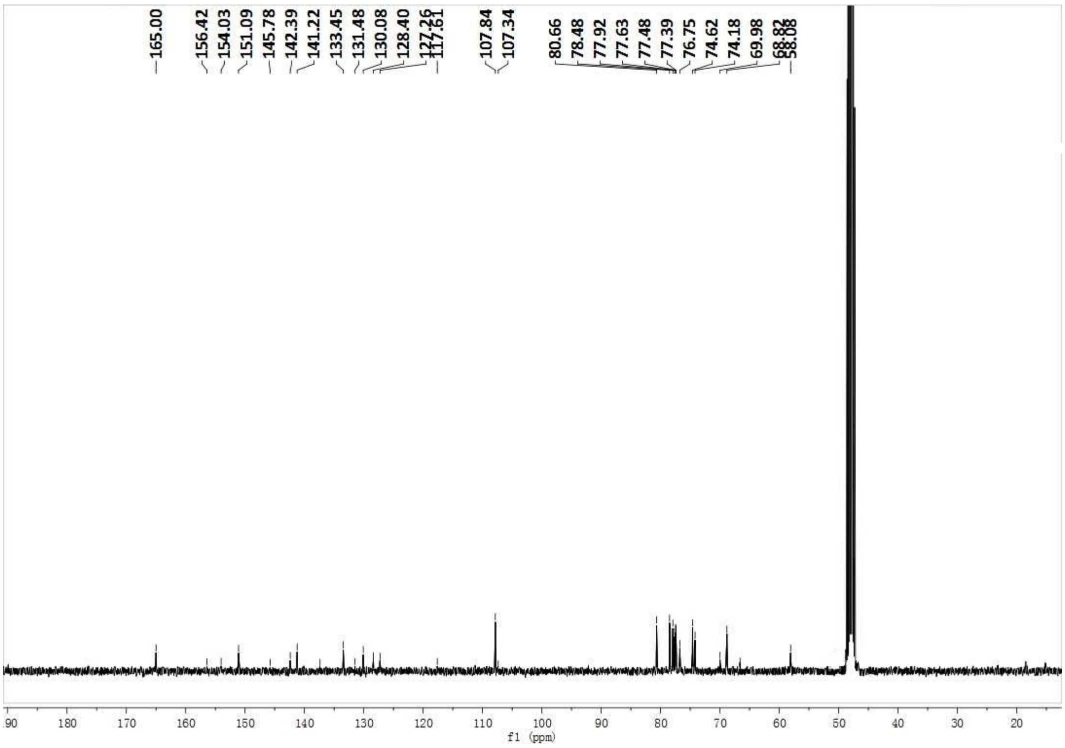

^{1}H NMR of **ZBW2'**:

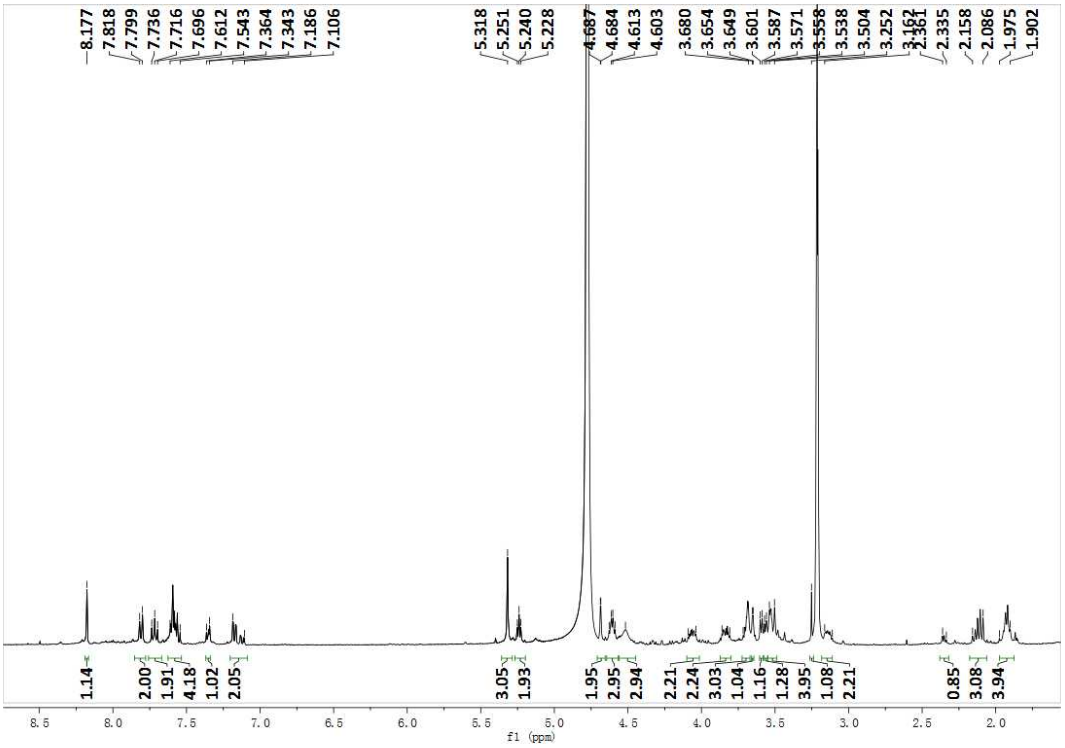

^{13}C NMR of **ZBW2'**:

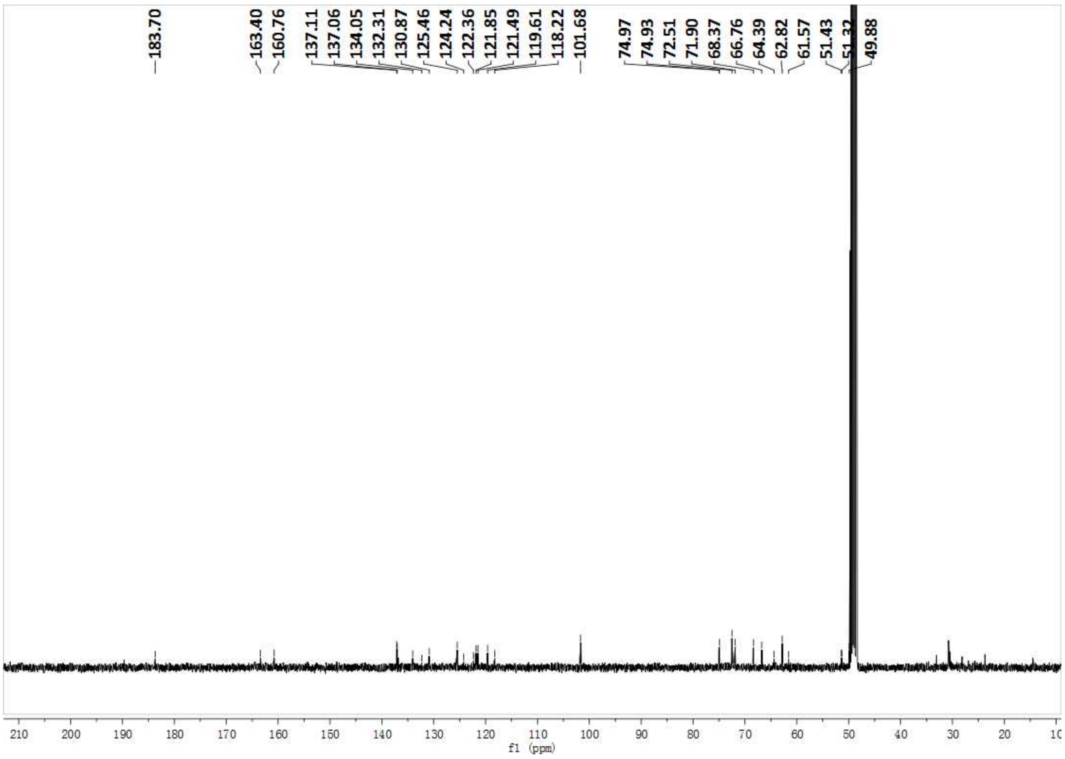

Supplement: Supplementary file 1 [file SC-006-C4SC03614J-s001.pdf]
